# Artificial sgRNAs engineered for genome editing with new Cas12b orthologs

**DOI:** 10.1038/s41421-019-0091-0

**Published:** 2019-04-23

**Authors:** Fei Teng, Tongtong Cui, Qingqin Gao, Lu Guo, Qi Zhou, Wei Li

**Affiliations:** 1 State Key Laboratory of Stem Cell and Reproductive Biology, Institute of Zoology, Chinese Academy of Sciences, 100101 Beijing, China; 2 Institute for Stem Cell and Regeneration, Chinese Academy of Sciences, 100101 Beijing, China; 3 University of Chinese Academy of Sciences, 100049 Beijing, China

**Keywords:** Biological techniques, Molecular biology

Dear Editor,

Precise genome editing is a promising area for its bright prospects in gene therapy, especially with the emergence of CRISPR-Cas (Clustered Regularly Interspaced Short Palindromic Repeats-CRISPR-associated proteins) systems. Up to now, three types of CRISPR-Cas systems, type II Cas9^1,2^, type V-A Cas12a^3^, and type V-B Cas12b^4^, have been successfully harnessed to facilitate mammalian genome editing. Among these, the relatively smaller size and higher specificity make Cas12b an ideal choice for in vivo gene therapy. However, only two Cas12b proteins (AaCas12b and AkCas12b) have been engineered for mammalian genome editing^4^, partly due to the lack of sequence information of the CRISPR array. Previous studies indicated that the RNA and protein components were interchangeable between closely related Cas9 systems^5^ as well as Cas12a systems^3^, and could be preliminarily optimized^6,7^, suggesting the possibility of synthesizing an artificial guide RNA scaffold interchangeable among different CRISPR effector proteins. Here, we explore the possibilities of expanding the Cas12b toolbox to additional orthologs and uncover mechanisms underlying Cas12b-mediated DNA cleavage. We not only demonstrate the interchangeability of dual-RNA:Cas12b in eight CRISPR-Cas12b systems, but also show that sequence- and structure-conserved artificial sgRNAs could enable Cas12b orthologs without known CRISPR array to achieve efficient genome editing in human cells.

To explore new Cas12b orthologs, we synthesized four unreported Cas12b-family proteins from diverse bacteria (BhCas12b, Bs3Cas12b, LsCas12b, and SbCas12b) as well as four previously reported Cas12b orthologs (AaCas12b, AkCas12, AmCas12b, and BsCas12b) to conduct genome editing in human embryonic kidney 293T cells^4^ (Fig. 1a, Supplementary Figs. S1a, b and S2, Supplementary Table S1 and Supplementary Sequences). To conduct mammalian genome editing, we co-transfected 293T cells with individual Cas12b enzymes and their cognate chimeric single-guide RNAs (sgRNAs) targeting human endogenous loci containing appropriate PAMs (Supplementary Fig. S1a, b, Supplementary Tables S2 and S3, and Supplementary Sequences). Results of T7 Endonuclease I (T7EI) assay showed that apart from previously reported AaCas12b and AkCas12b^4^, four novel Cas12b orthologs (AmCas12b, BhCas12b, Bs3Cas12b, and LsCas12b) could also edit the human genome, although their targeting efficiencies varied at different targeting sites (Fig. 1b and Supplementary Fig. S3a). We also achieved multiplex genome engineering by programming Bs3Cas12b to simultaneously edit four sites in the human genome by simply using multiple sgRNAs (Supplementary Fig. S3b, c). These newly identified Cas12b orthologs expand our options for Cas12b-based genome engineering.

**Fig. 1:**
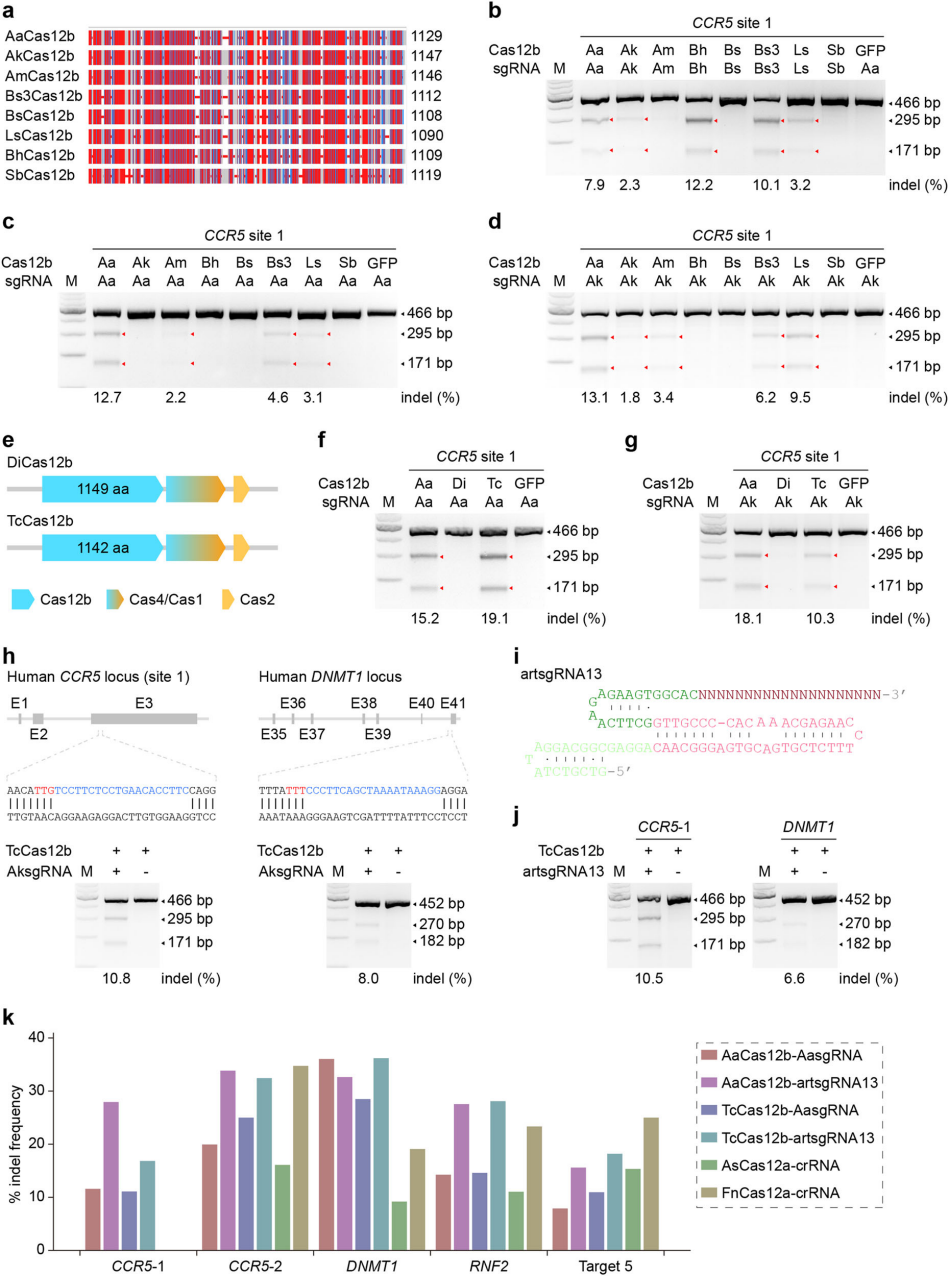
Orthologous Cas12b proteins for artificial RNA-guided genome editing. **a** Graphical overview of the eight Cas12b orthologs tested in this study. Sizes (amino acids) are indicated. **b** T7EI assay results indicating the genome targeting activities of the eight Cas12b orthologs directed by their cognate sgRNAs in human 293T cells. Red triangles indicate the cleaved bands. **c**, **d** T7EI assay results indicating the genome targeting activities of the eight Cas12b orthologs directed by AasgRNA (**c**) and AksgRNA (**d**) in human 293T cells. Red triangles indicate the cleaved bands. **e** Maps of bacterial genomic loci corresponding to DiCas12b and TcCas12b. The two Cas12b loci have no CRISPR array. **f**, **g** T7EI assay results indicating the genome targeting activities of AaCas12b, DiCas12b, and TcCas12b directed by AasgRNA (**f**) and AksgRNA (**g**) in human 293T cells. Red triangles indicate the cleaved bands. **h** T7EI assay results indicating the simultaneous multiplex genome targeting mediated by TcCas12b combined with AksgRNAs in human 293T cells. **i** Schematic illustration of the secondary structures of artificial sgRNA scaffold 13 (artsgRNA13). **j** T7EI assay results indicating the simultaneous multiplex genome targeting mediated by TcCas12b combined with artsgRNA13s in human 293T cells. **k** T7EI analysis of average targeted mutation efficiencies induced at five different endogenous loci by AaCas12b, TcCas12b, AsCas12a, and FnCas12a. *n* = 2

Of interest, we found that the amino acid sequences of these Cas12b orthologs are highly conserved (Fig. 1a and Supplementary Fig. S2). Meanwhile, the DNA sequences of pre-crRNA:tracrRNA duplex (Supplementary Fig. S4) and their secondary structures (Supplementary Fig. S1b) were also highly conserved. These results reminded us that the interchangeability of the RNA:Cas components in Cas9 and Cas12a systems^3,5^ might also exist in Cas12b systems. To investigate the interchangeability of the two components in Cas12b systems, we conducted genome editing in 293T cells with individual Cas12b orthologs complexed with cognate and non-cognate sgRNAs originated from the eight systems. As results of T7EI assay indicated, non-cognate sgRNAs derived from AaCas12b, AkCas12b, AmCas12b, Bs3Cas12b, and LsCas12b loci could substitute the cognate sgRNAs for mammalian genome editing, although their activities varied between different Cas12b orthologs and sgRNAs (Fig. 1c, d and Supplementary Fig. S5a–e). These results demonstrated that the Cas12b effectors could work with other system-derived dual-RNAs to effectively cut the target DNA and thus the RNA:Cas12b components are interchangeable in the investigated systems.

Based on the above findings, we proposed a hypothesis that the well-known non-cognate sgRNAs might be sufficient to facilitate Cas12b orthologs without known CRISPR arrays to achieve genome editing. To test this hypothesis, we first synthesized two Cas12b orthologs from *D. inopinatus* (DiCas12b) and *T. calidus* (TcCas12b), which were considered unsuitable for genome-editing applications as the sequences of their crRNA:tracrRNA duplex were unpredictable (Fig. 1e, Supplementary Table S1, and Supplementary Sequences). Then we co-transfected DiCas12b, TcCas12b, or AaCas12b combined with the sgRNAs derived from loci of the eight Cas12b orthologs (Supplementary Figure S1b) targeting different genomic sites in 293T cells. T7EI assay results indicated that sgRNAs derived from AaCas12b, AkCas12b, AmCas12b, Bs3Cas12b, and LsCas12b could enable TcCas12b to robustly edit the human genome (Fig. 1f, g and Supplementary Fig. S6a–f). Furthermore, TcCas12b could facilitate multiplex genome editing simultaneously directed by either AasgRNAs or AksgRNAs (Fig. 1h and Supplementary Fig. S7a–c). These results above demonstrated that the interchangeability we identified of Cas12b and dual-RNA from different systems could empower the Cas12b ortholog without known CRISPR array to functionally target and cleave the mammalian genomes.

The exchangeability of Cas12b and dual-RNA in Cas12b systems further promoted us to design new artificial sgRNA (artsgRNA) scaffolds to facilitate Cas12b-mediated genome editing. Considering the conservation of DNA sequences and secondary structures among Cas12b orthologs (Supplementary Figs. S1b and S4), we designed and de novo synthesized 37 sgRNA scaffolds targeting human *CCR5* locus (Fig. 1i, Supplementary Fig. S8a, and Supplementary Table S4), which contained various base substitutions whilst maintained the correct secondary structure. The results of T7EI assay suggested that 22 artsgRNA scaffolds could work efficiently (Supplementary Fig. S8b). To test the generalization of our artsgRNAs in facilitating Cas12b-mediated genome editing, we performed multiplex genome editing using TcCas12b or AaCas12b directed by artsgRNA13 (Fig. 1i), one of the most efficient artsgRNAs in our test (Supplementary Fig. S8b). T7EI assay results indicated that artsgRNA13s could simultaneously facilitate multiplex genome engineering with both TcCas12b and AaCas12b (Fig. 1j and Supplementary Fig. S8c). Furthermore, we compared the frequencies of targeted mutations induced by TcCas12b, AaCas12b^4^, AsCas12a^3^, and FnCas12a^3^ at five chromosomal target sites in human 293T cells (Supplementary Table S3). The results of T7EI assay indicated that artsgRNA13 could enhance the targeting efficiencies of both TcCas12b and AaCas12b to reach a similar efficiency of FnCas12a, which was higher than the efficiency of AsCas12a (Fig. 1k). Our results suggested that we could design and synthesize artsgRNAs to facilitate Cas12b-mediated multiplex genome editing.

In summary, we engineered a series of Cas12b orthologs for genome editing in mammalian cells, which expanded the Cas12b-based genome-engineering toolbox. By providing the first experimental evidence enlightening the exchangeability of the two core components in type V-B CRISPR system, the dual-RNA and the effector protein, we successfully harness TcCas12b whose bacterial locus harbors no known CRISPR array for robust mammalian genome editing using orthologous sgRNAs. Importantly, our findings enlighten principles of RNA-component design which could facilitate Cas12b nucleases even without a CRISPR array to conduct precise and multiplex genome engineering. Although the exact bases in the sgRNA scaffolds responsible for the cleavage capacity of Cas12b-RNA-DNA complexes are not yet elucidated and more structural characterizations are still required, we believe that these insights will largely facilitate the mechanistic research and broader applications of CRISPR systems, and shed light on de novo synthesis of enzymatic genome-editing systems.

## Supplementary information

Supplementary Information

Supplementary, Table S1

Supplementary, Table S2

Supplementary, Table S4

## Supplementary information

**Supplementary Information** accompanies the paper at (10.1038/s41421-019-0091-0).
