# Supplementary material for: Artificial sgRNAs engineered for genome editing with new Cas12b orthologs
Source: Cell Discov. 2019 Apr 23;5:23. doi: 10.1038/s41421-019-0091-0 (PMC6476878; doi:10.1038/s41421-019-0091-0)
Supplement: Supplementary file 1 — Supplementary Information [file 41421_2019_91_MOESM1_ESM.pdf]

Supplementary Information

Materials and Methods

DNA manipulations. DNA manipulation including DNA preparation, digestion, ligation, amplification, synthesis, purification, agarose gel electrophoresis, *etc.* were conducted according to *Molecular Cloning: A Laboratory Manual* with some modifications. Briefly, targeting chimeric single guide RNA (sgRNA) scaffolds for cell transfection assay were constructed by ligation annealed oligonucleotides (oligos) (Supplementary Table S3) into BsaI-digested pUC19-U6-sgRNA vectors (Supplementary Sequences) or directly *de novo* synthesized (Supplementary Table S4) into pUC19-U6 vector[1].

***De novo* gene synthesis and plasmid construction.** PSI-BLAST program[2] was adopted to identify new CRISPR-Cas12b orthologs. Their coding sequences were humanized[3] and oligos for Cas12b gene and sgRNA synthesis were designed using GeneDesign program[4]. All oligos for gene synthesis were commercially purchased (Taihe Biotechnology Co., LTD). For Cas12b coding genes which are >3 kb were split into 4 "chunks" of ~800 bp (Supplementary Table S1). For gRNAs which are <300 bp were left as is (Supplementary Tables S2 and S4). All oligos for gene synthesis were commercially purchased (Taihe Biotechnology Co., LTD). DNA fragments were synthesized using overlap extension PCR method according to our previous study[1]. Purified products were assembled into expression vectors via homologous recombination in vitro using NEBuilder® HiFi DNA Assembly Master Mix (NEB). The pCAG-2AeGFP and pUC19-U6 vectors[1] were applied for mammalian expression of Cas12b proteins and sgRNAs, respectively. AaCas12b, AkCas12b, AmCas12b, BsCas12b and their cognate sgRNAs have been previously reported[1].

Cell culture and transfection. Human embryonic kidney 293T cells were cultured in Dulbecco's Modified Eagle's Medium (DMEM, Gibco) supplemented with 10% fetal bovine serum (FBS, Gibco) and 1% Antibiotic-Antimycotic (Gibco) at 37°C with 5% CO_2 incubation. 293T cells were transfected using Lipofectamine LTX (Invitrogen) following the manufacturer's recommended protocol. For each well of a 48-well plate, a total of 400 ng

plasmid (Cas12b: sgRNA = 2: 1) were used. Then 48 h following transfection, cells were harvested directly for genomic DNA extraction without sorting.

T7 endonuclease I (T7EI) assay and Sanger sequencing. Harvested cells were lysed directly with Buffer L (Bimake) supplemented with Protease K and incubated at 55℃ for 3 h and inactivated at 95℃ for 10 min. Genomic region surrounding the Cas12b target site for each gene was PCR-amplified (Supplementary Table S5). 200 ~ 400 ng PCR products were mixed with ddH_2O to a final volume of 10 μL, and subjected to re-annealing process to enable heteroduplex formation according to previous methods[5]. After re-annealing, products were treated with 1/10 volume of NEBuffer™ 2.1 and 0.2 μL T7EI (NEB) at 37 ℃ for 30 min, and analyzed on 3% agarose gels. Indels were quantitated based on relative band intensities[6]. T7EI assay identified mutated products were subjected to be cloned into TA cloning vector and transformed into competent *E. coli* strain (Transgen Biotech). After overnight culture, colonies were randomly picked out and sequenced.

For each sample, transfection and T7EI assay were repeated twice or more and a representative result was shown. The histogram in this study represented the mean value of two independent repeats.

References

1. Teng, F. et al. Repurposing CRISPR-Cas12b for mammalian genome engineering. *Cell Discov.* **4**, 63 (2018).
2. Altschul, S.F. et al. Gapped BLAST and PSI-BLAST: a new generation of protein database search programs. *Nucleic Acids Res.* **25**, 3389-3402 (1997).
3. Grote, A. et al. JCat: a novel tool to adapt codon usage of a target gene to its potential expression host. *Nucleic Acids Res.* **33**, W526-531 (2005).
4. Richardson, S.M., Wheelan, S.J., Yarrington, R.M. & Boeke, J.D. GeneDesign: rapid, automated design of multikilobase synthetic genes. *Genome Res.* **16**, 550-556 (2006).
5. Li, W., Teng, F., Li, T. & Zhou, Q. Simultaneous generation and germline transmission of multiple gene mutations in rat using CRISPR-Cas systems. *Nat. Biotechnol.* **31**, 684-686 (2013).
6. Cong, L. et al. Multiplex genome engineering using CRISPR/Cas systems. *Science* **339**, 819-823 (2013).

Figure S1

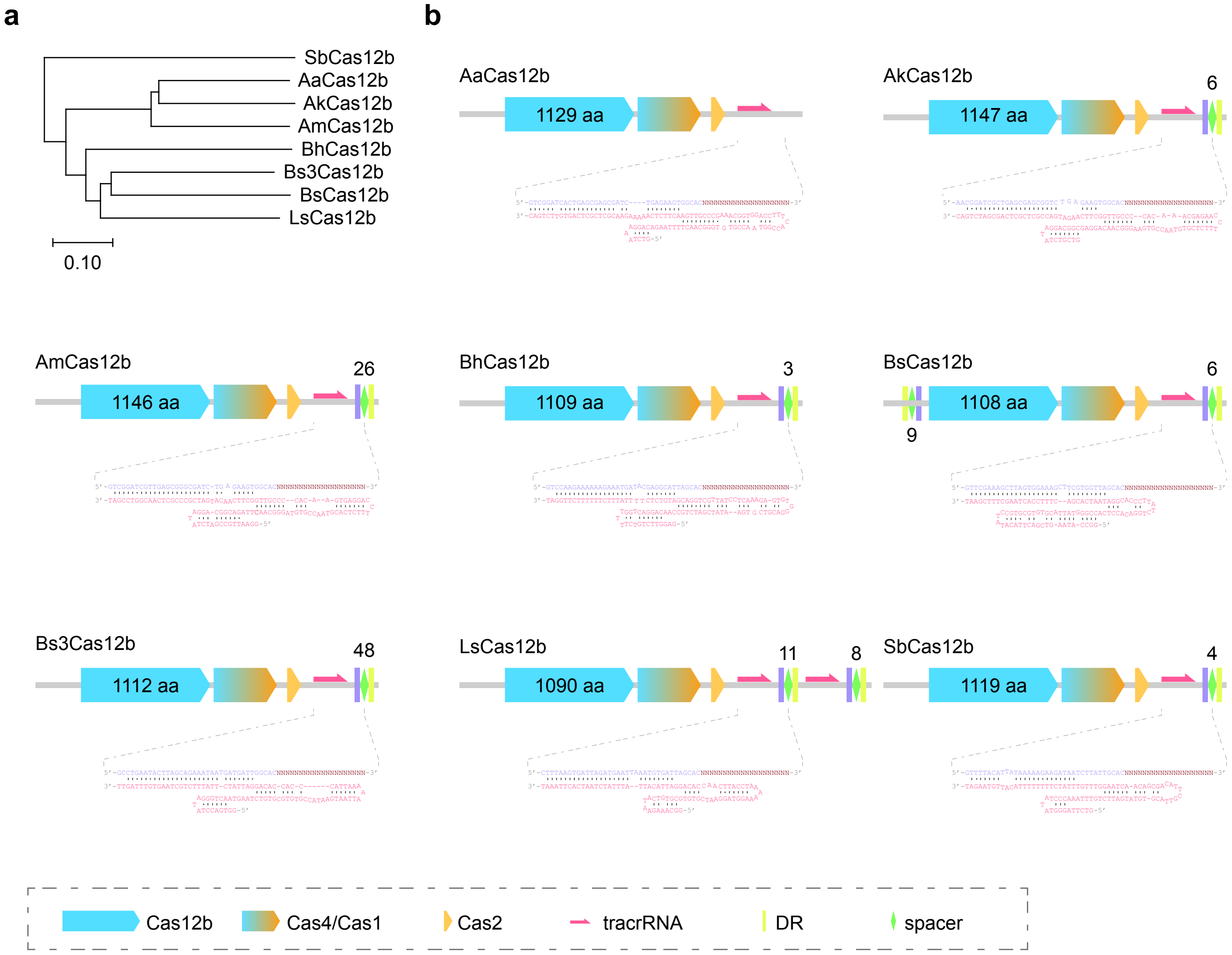
a
SbCas12b
AaCas12b
AkCas12b
AmCas12b
BhCas12b
Bs3Cas12b
BsCas12b
LsCas12b
0.10
b
AaCas12b
1129 aa
AkCas12b
1147 aa
6
AmCas12b
1146 aa
26
BhCas12b
1109 aa
3
BsCas12b
1108 aa
6
9
Bs3Cas12b
1112 aa
48
LsCas12b
1090 aa
11
8
SbCas12b
1119 aa
4
Cas12b
Cas4/Cas1
Cas2
tracrRNA
DR
spacer

Figure S1 Phylogenetic tree of non-redundant Cas12b orthologs and their loci chosen for genome-editing testing. a Neighbor Joining phylogenetic tree showing the evolutionary relationships of Cas12b orthologs tested in this study. **(b)** Maps of bacterial genomic loci corresponding to the eight Cas12b proteins in Supplementary Fig. S1a. In silico co-folding of the crRNA DR and putative tracrRNA shows stable secondary structure. DR, direct repeat. The number of each bacterial genomic spacers is indicated above their CRISPR array.

Figure S2

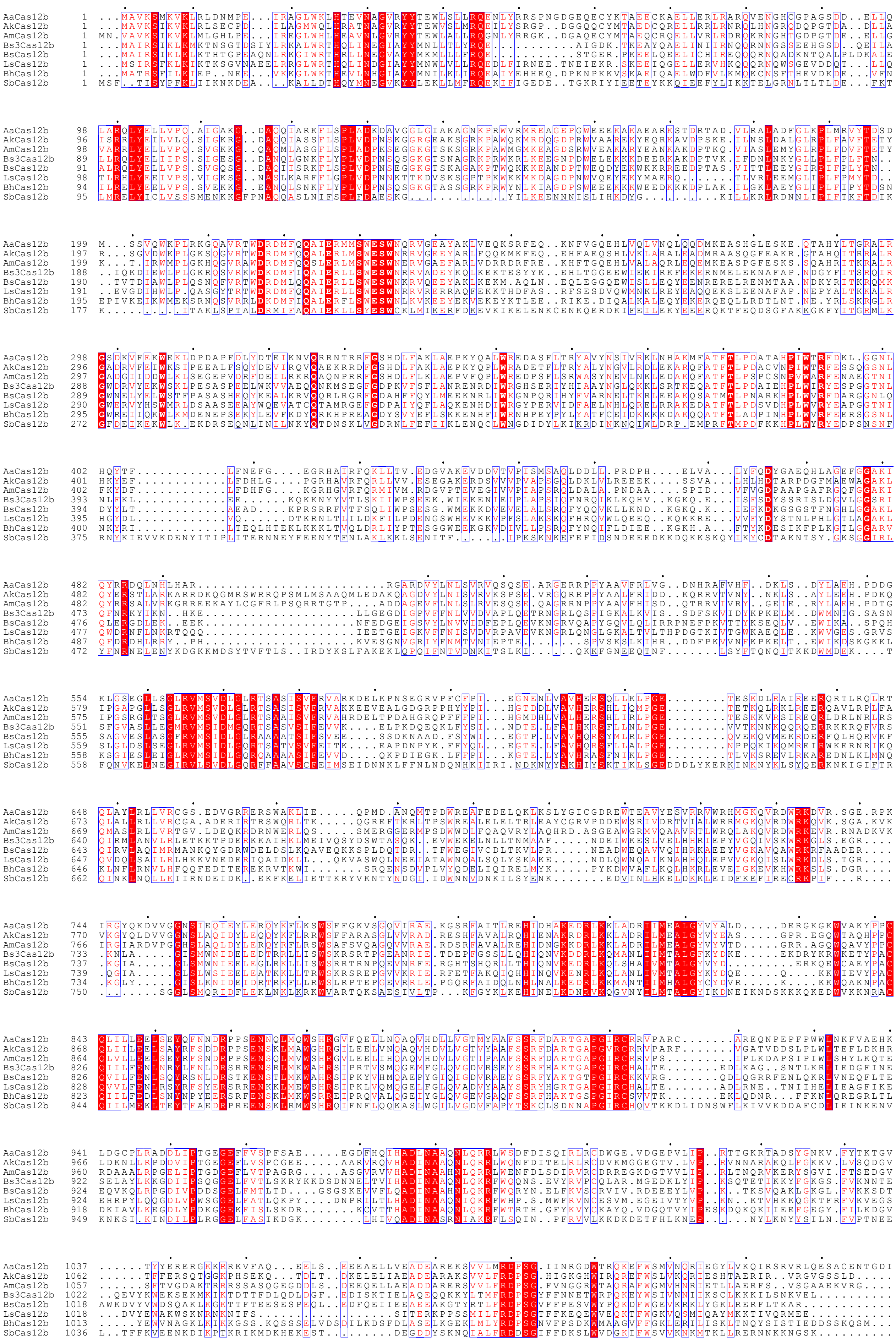
AaCas12b
AkCas12b
AmCas12b
Bs3Cas12b
BsCas12b
LsCas12b
BhCas12b
SbCas12b

Figure S2 Protein alignment of Cas12b orthologs. Multiple sequence alignment of the amino acid sequences of the eight Cas12b orthologs tested in this study. Residues that are conserved are highlighted with a red background and conserved mutations are highlighted with an outline and red font.

Figure S3

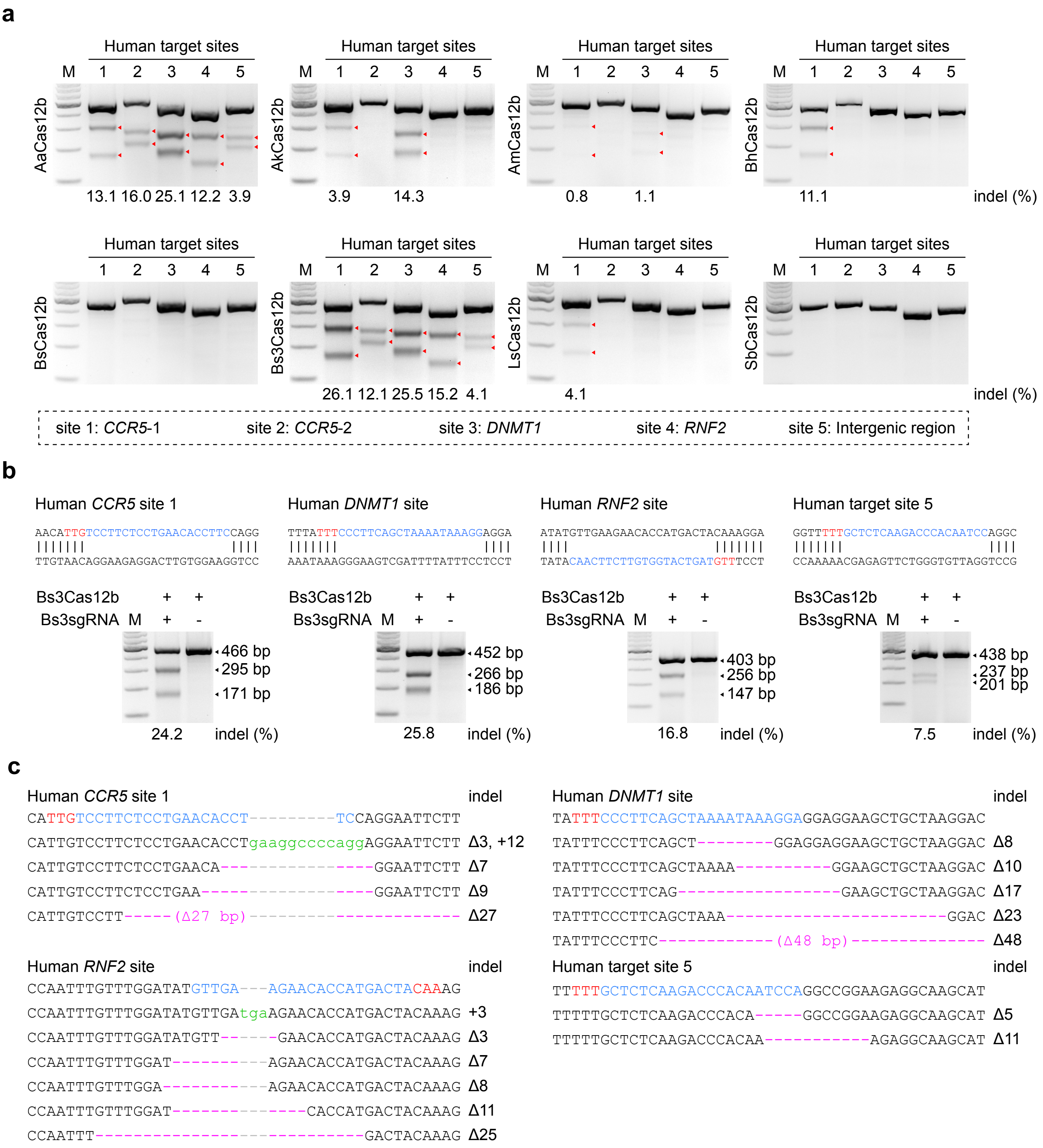

a
Human target sites
M 1 2 3 4 5
AaCas12b
13.1 16.0 25.1 12.2 3.9
AkCas12b
3.9 14.3
AmCas12b
0.8 1.1
BhCas12b
11.1
indel (%)
BsCas12b
Bs3Cas12b
26.1 12.1 25.5 15.2 4.1
LsCas12b
4.1
SbCas12b
site 1: CCR5-1
site 2: CCR5-2
site 3: DNMT1
site 4: RNF2
site 5: Intergenic region
b
Human CCR5 site 1
AACATTGTCCTTCTCCTGAACACCTTCCAGG
TTGTAACAGGAAGAGGACTTGTGGAAGGTCC
Human DNMT1 site
TTTATTTCCCTTCAGCTAAAATAAAGGAGGA
AAATAAAGGGAAGTCGATTTTATTTCCTCCT
Human RNF2 site
ATATGTTGAAGAACACCATGACTACAAAGGA
TATACAACTTCTTGTGGTACTGATGTTTCCT
Human target site 5
GGTTTTTGCTCTCAAGACCCACAATCCAGGC
CCAAAAACGAGAGTTCTGGGTGTTAGGTCCG
Bs3Cas12b + +
Bs3sgRNA M + -
466 bp
295 bp
171 bp
24.2
452 bp
266 bp
186 bp
25.8
403 bp
256 bp
147 bp
16.8
438 bp
237 bp
201 bp
7.5
indel (%)
c
Human CCR5 site 1 indel
CATTGTCCTTCTCCTGAACACCT---------TCCAGGAATTCTT
CATTGTCCTTCTCCTGAACACCTgaaggccccaggAGGAATTCTT Δ3, +12
CATTGTCCTTCTCCTGAACA----------------GGAATTCTT Δ7
CATTGTCCTTCTCCTGAA------------------GGAATTCTT Δ9
CATTGTCCTT-----(Δ27 bp)---------------------- Δ27
Human DNMT1 site indel
TATTTCCCTTCAGCTAAAATAAAGGAGGAGGAAGCTGCTAAGGAC
TATTTCCCTTCAGCT--------GGAGGAGGAAGCTGCTAAGGAC Δ8
TATTTCCCTTCAGCTAAAA----------GGAAGCTGCTAAGGAC Δ10
TATTTCCCTTCAG-----------------GAAGCTGCTAAGGAC Δ17
TATTTCCCTTCAGCTAAA-----------------------GGAC Δ23
TATTTCCCTTC------------(Δ48 bp)-------------- Δ48
Human RNF2 site indel
CCAATTTGTTTGGATATGTTGA---AGAACACCATGACTACAAAG
CCAATTTGTTTGGATATGTTGAtgaAGAACACCATGACTACAAAG +3
CCAATTTGTTTGGATATGTT------GAACACCATGACTACAAAG Δ3
CCAATTTGTTTGGAT----------AGAACACCATGACTACAAAG Δ7
CCAATTTGTTTGGA-----------AGAACACCATGACTACAAAG Δ8
CCAATTTGTTTGGAT--------------CACCATGACTACAAAG Δ11
CCAATTT----------------------------GACTACAAAG Δ25
Human target site 5 indel
TTTTTGCTCTCAAGACCCACAATCCAGGCCGGAAGAGGCAAGCAT
TTTTTGCTCTCAAGACCCACA-----GGCCGGAAGAGGCAAGCAT Δ5
TTTTTGCTCTCAAGACCCACAA-----------AGAGGCAAGCAT Δ11

Figure S3 Cas12b orthologs mediated genome targeting in human 293T cells. a T7EI assay results indicating the genome targeting activities of the eight Cas12b proteins combined with their cognate sgRNAs in the human genome, respectively. Red triangles indicate the cleaved bands. **b** T7EI assay results indicating the simultaneous multiplex genome targeting mediated by Bs3Cas12b combined with its cognate sgRNAs (Bs3sgRNAs) in human 293T cells. **c** Sanger sequencing showing representative indels induced by Bs3Cas12b combined with Bs3sgRNAs in Supplementary Fig. S3b. PAM and protospacer sequences are colored in red and blue, respectively. Deletions and insertions are symbolled with purple dashes and green lowercases, respectively.

Figure S4

```
                                                   tracrRNA
AasgRNA    1   ..........GTCTAAAGGACAGAATTTTTCAACGGGTGTGCCAATGGCCACTTTCCAGGTG..GCAAAGCCCGTTG
AksgRNA    1   .......GTCGTCTATAGGACGGCGAGGACAACGGGAAGTGCCAATGTGCTCTTTCCAAGAG..CAAACACCCCGTT
AmsgRNA    1   GGAATTGCCGATCTATAGGACGGCAGATTCAACGGGATGTGCCAATGCACTCTTTCCAGGAG..TGAACACCCCGTT
BhsgRNA    1   ......GAGGTTCTGTCTTTTGGTCAGGACAACCGTCTAGCTATAAGTGCTGCAGGGTGTGA....GAAACTCCTAT
BssgRNA    1   ............CCATAAG......TCGACTTACATATCCGTGCGTGTGCATTATGGGCCCATCCACAGGTCTATTC
Bs3sgRNA   1   ......GGTGACCTATAG.........GGTCAATGAATCTGTGCGTGTGCCATAAGTAATTA.....AAAATTACCC
LssgRNA    1   ...........................GGCAAAGAATACTGTGCGTGTGCTAAGGATGGAAA.....AAATCCATTC
SbsgRNA    1   ...........GTCTTAGGGTATATCCCAAATTTGTCTTAGTATGTGCATTGCTTACAGCGA..............C

                         tracrRNA                                 crRNA
AasgRNA    66  AACTTCTCAAAAAGAACGCTCG....CTCAGTGTTCTGACGTCGGATCACTGAGCGAGCGATCTGAGAAGTGGCAC
AksgRNA    69  GGCT..TCAAGATGACCGCTCG....CTCAGCGATCTGACAACGGATCGCTGAGCGAGCGGTCTGAGAAGTGGCAC
AmsgRNA    76  GGCT..TCAACATGATCGCCCG....CTCAACGGTCCGATGTCGGATCGTTGAGCGGGCGATCTGAGAAGTGGCAC
BhsgRNA    68  TGCT..GGACGATGTCTCTTTTATTTCTTTTTTCTTGGATGTCCAAGAAAAAAGAAATGATACGAGGCATTAGCAC
BssgRNA    60  CCAC..GGATAATCACGACTTTCCA.CTAAGCTTTCGAATGTTCGAAAGCTTAGTGGAAAGCTTCGTGGTTAGCAC
Bs3sgRNA   58  ACC...ACAGGATTATCTTATTTCTGCTAAGTGTTTAGTTGCCTGAATACTTAGCAGAAATAATGATGATTGGCAC
LssgRNA    46  AACC..ACAGGATTACATTATTTAT.CTAATCACTTAAATCTTTAAGTGATTAGATGAATTAAATGTGATTAGCAC
SbsgRNA    53  AACT..AAGGTTTGTTTATCTTTTTTTTACATTGTAAGATGTTTTACATTATAAAAAGAAGATAATCTTATTGCAC
```

Figure S4 DNA alignment of sgRNAs of Cas12b. Multiple sequence alignment of the DNA sequences of the 8 sgRNAs derived from the 8 Cas12b loci tested in this study.

Figure S5

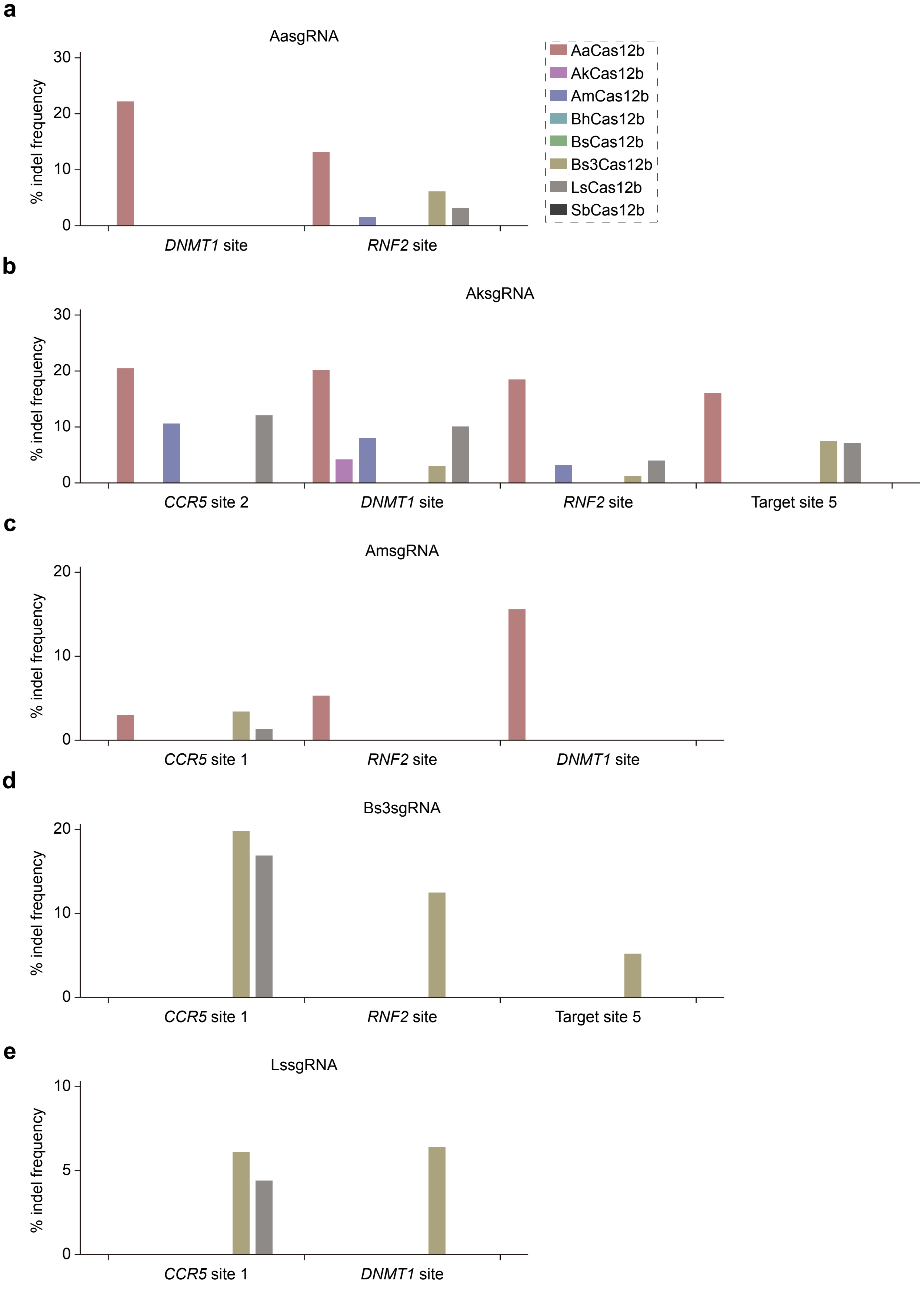

a
AasgRNA
% indel frequency
30
20
10
0
DNMT1 site
RNF2 site
AaCas12b
AkCas12b
AmCas12b
BhCas12b
BsCas12b
Bs3Cas12b
LsCas12b
SbCas12b
b
AksgRNA
% indel frequency
30
20
10
0
CCR5 site 2
DNMT1 site
RNF2 site
Target site 5
c
AmsgRNA
% indel frequency
20
10
0
CCR5 site 1
RNF2 site
DNMT1 site
d
Bs3sgRNA
% indel frequency
20
10
0
CCR5 site 1
RNF2 site
Target site 5
e
LssgRNA
% indel frequency
10
5
0
CCR5 site 1
DNMT1 site

Figure S5 Interchangeability between Cas12b orthologs and their sgRNAs. T7EI assay results indicating the average genome targeting activities of the eight Cas12b orthologs directed by AasgRNA **(a)**, AksgRNA **(b)**, AmsgRNA **(c)**, Bs3sgRNA **(d)** and LssgRNA **(e)** in human 293T cells, respectively. Red triangles indicate the cleaved bands. n = 2.

Figure S6

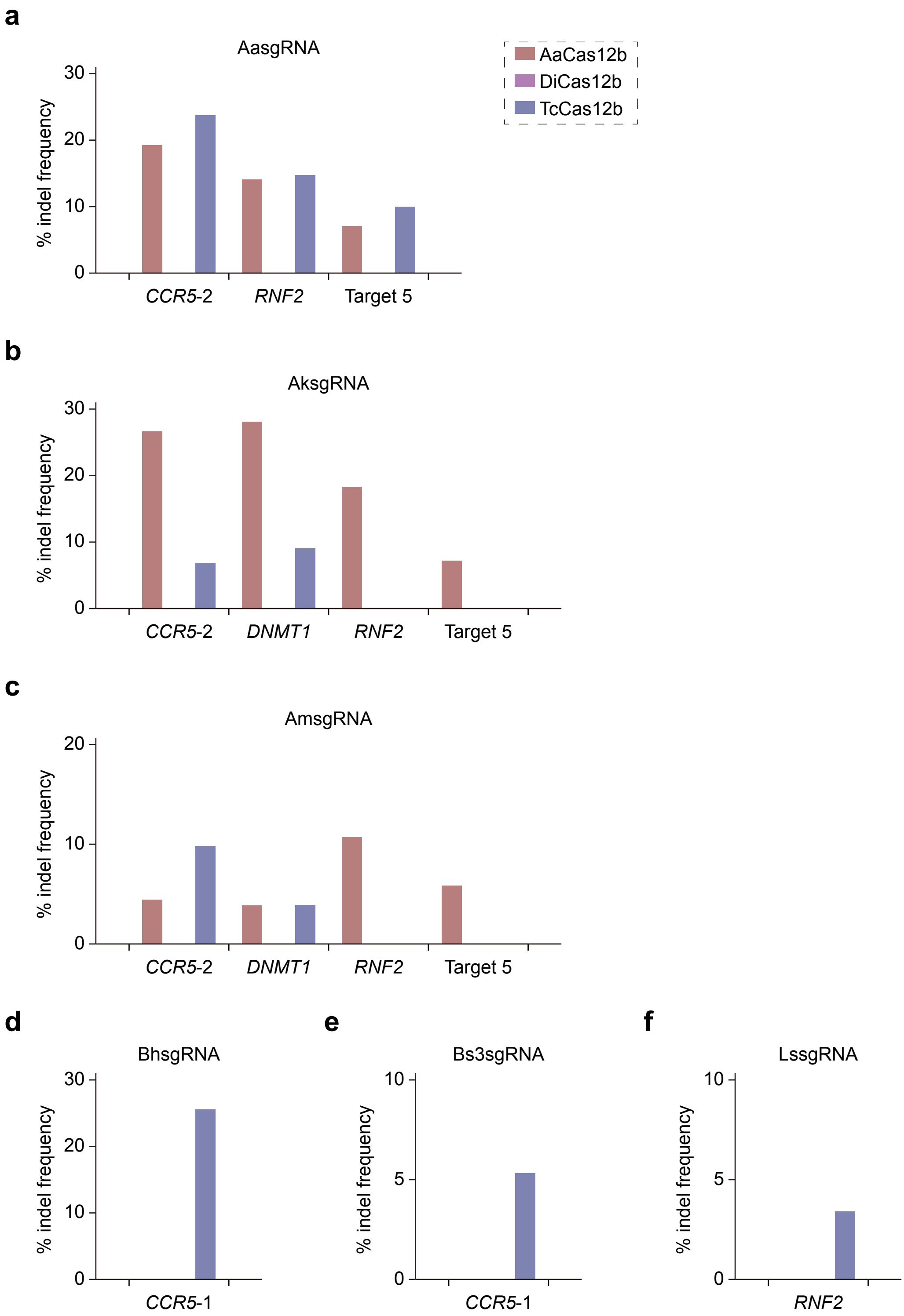

a
AasgRNA
AaCas12b
DiCas12b
TcCas12b
% indel frequency
30
20
10
0
CCR5-2
RNF2
Target 5
b
AksgRNA
% indel frequency
30
20
10
0
CCR5-2
DNMT1
RNF2
Target 5
c
AmsgRNA
% indel frequency
20
10
0
CCR5-2
DNMT1
RNF2
Target 5
d
BhsgRNA
% indel frequency
30
20
10
0
CCR5-1
e
Bs3sgRNA
% indel frequency
10
5
0
CCR5-1
f
LssgRNA
% indel frequency
10
5
0
RNF2

Figure S6 Orthologous sgRNAs directed Cas12b for genome editing. T7EI assay results indicating the average genome targeting activities of AaCas12b, DiCas12b and TcCas12b directed by AasgRNA **(a)**, AksgRNA **(b)**, AmsgRNA **(c)**, BhsgRNA **(d),** Bs3sgRNA **(e)** and LssgRNA **(f)** in the human 293T cells, respectively. Red triangles indicate the cleaved bands. n = 2.

Figure S7

a

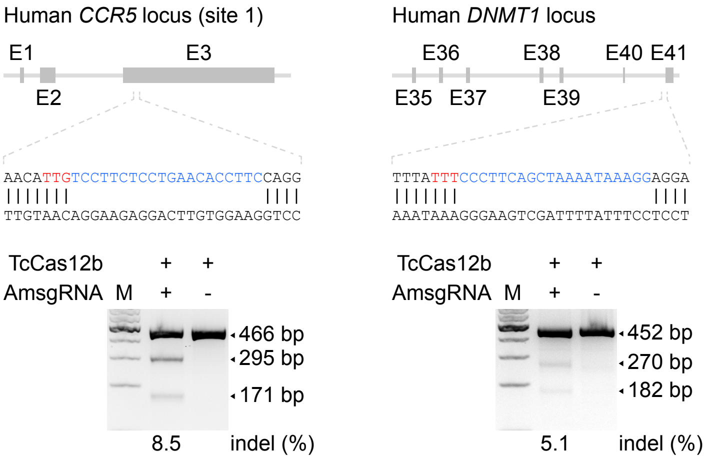

b

```
AksgRNA-CCR5-1                                                     indel
TACAACATTGTCCTTCTCCTGAACACCTTCCAGGAATTCTTTGGCCTGAATAATTGCAGT
TACAACATTGTCCTTCTCCTGAACACCT------AATTCTTTGGCCTGAATAATTGCAGT  Δ6
TACAACATTGTCCTTCTCCTGAACA------------TCTTTGGCCTGAATAATTGCAGT  Δ12
TACAACATTGTCCTTCTCCTGAA---------------------------TAATTGCAGT  Δ27
AksgRNA-DNMT1                                                      indel
CCTTTTATTTCCCTTCAGCTAAAATAAAGGAGGAGGAAGCTGCTAAGGACTAGTTCTGCC
CCTTTTATTTCCCTTCAGCTAAA-----GGAGGAGGAAGCTGCTAAGGACTAGTTCTGCC  Δ5
CCTTTTATTTCCCTTCAGCTAAAAa--------AGGAAGCTGCTAAGGACTAGTTCTGCC  Δ9, +1
CCTTTTATTTCCCTTCAGCTAAA-----------------TGCTAAGGACTAGTTCTGCC  Δ17
```

c

```
AmsgRNA-CCR5-1                                                     indel
TACAACATTGTCCTTCTCCTGAACACCTTCCAGGAATTCTTTGGCCTGAATAATTGCAGT
TACAACATTGTCCTTCTCCTGAACACCTga-AGGAATTCTTTGGCCTGAATAATTGCAGT  Δ3, +2
TACAACATTGTCCTTCTCCTGAACA---TCCAGGAATTCTTTGGCCTGAATAATTGCAGT  Δ3
TACAACATTGTCCTTCTCCTGAACACCga--AGGAATTCTTTGGCCTGAATAATTGCAGT  Δ4, +2
TACAACATTGTCCTTCTCCTGAACACCT----GGAATTCTTTGGCCTGAATAATTGCAGT  Δ4
TACAACATTGTCCTTCTCCTGAACACC-------AATTCTTTGGCCTGAATAATTGCAGT  Δ7
TACAACATTGTCCTTCTCCTGAA---------------------------TAATTGCAGT  Δ27
TACAACATTGTCCTTCTCC--------------------------------AATTGCAGT  Δ32
AmsgRNA-DNMT1                                                      indel
CCTTTTATTTCCCTTCAGCTAAAATAAAGGAGGAGGAAGCTGCTAAGGACTAGTTCTGCC
CCTTTTATTTCCCTTCAGCTAAAtata--GAGGAGGAAGCTGCTAAGGACTAGTTCTGCC  Δ6, +4
CCTTTTATTTCCCTTCAGCTAAAAT------GGAGGAAGCTGCTAAGGACTAGTTCTGCC  Δ6
CCTTTTATTTCCCTTCAGCTAAAg----------GGAAGCTGCTAAGGACTAGTTCTGCC  Δ11, +1
CCTTTTATTTCCCTTCAGCTAAA-----------GGAAGCTGCTAAGGACTAGTTCTGCC  Δ11
CCTTTTATTTCCCTTCAGCT---------------GAAGCTGCTAAGGACTAGTTCTGCC  Δ15
```

Figure S7 TcCas12b-mediated multiplex genome editing. a T7EI assay results indicating the simultaneous multiplex genome targeting mediated by TcCas12b combined with AmsgRNAs in human 293T cells. **b-c** Sanger sequencing showing representative indels induced by TcCas12b combined with AksgRNAs in Fig. 1h **(b)** and AmsgRNAs in Supplementary Fig. S7a **(c)**. PAM and protospacer sequences are colored in red and blue, respectively. Deletions and insertions are symbolled with purple dashes and green lowercases, respectively.

Figure S8

a

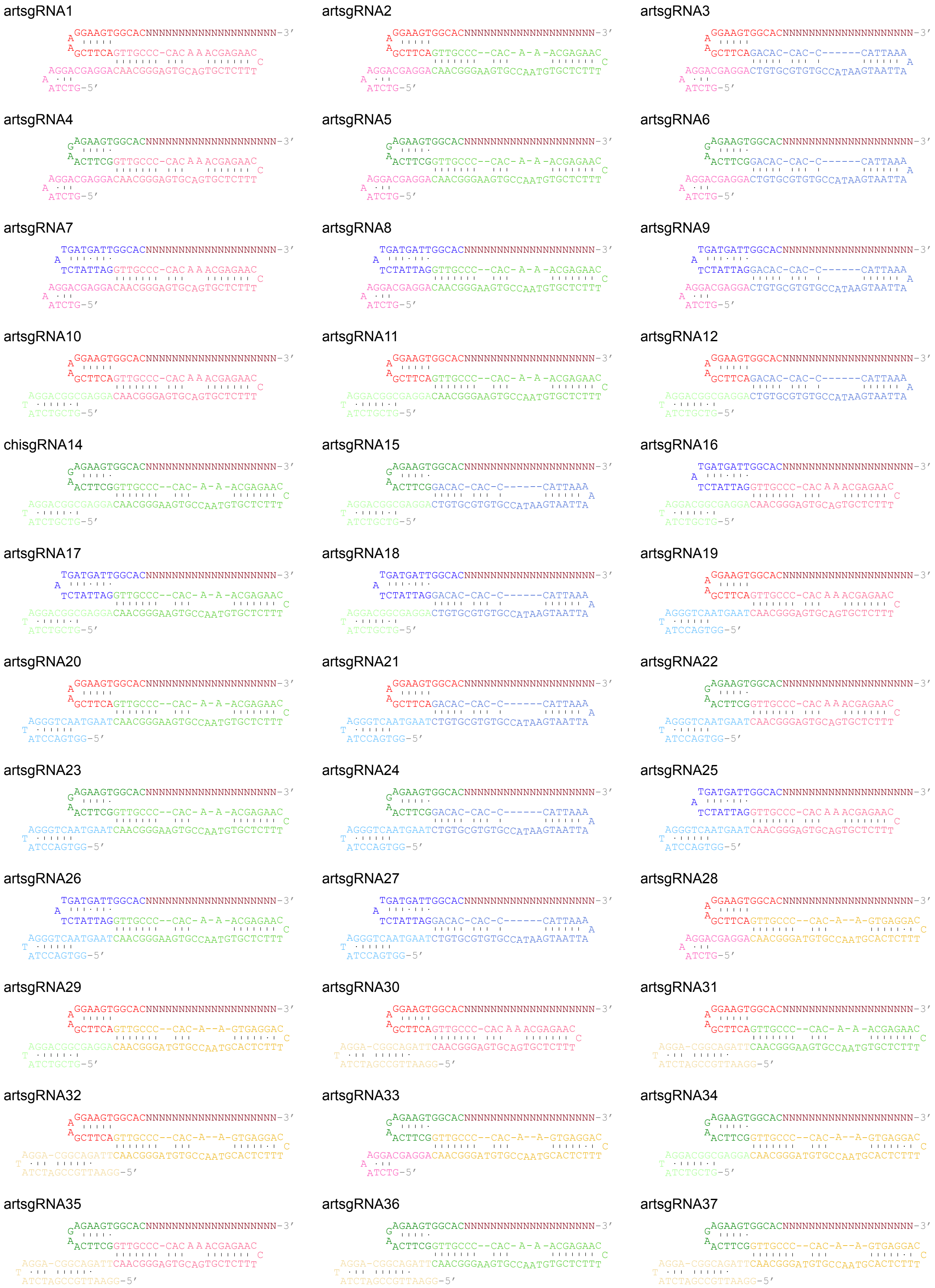

artsgRNA1
artsgRNA2
artsgRNA3
artsgRNA4
artsgRNA5
artsgRNA6
artsgRNA7
artsgRNA8
artsgRNA9
artsgRNA10
artsgRNA11
artsgRNA12
chisgRNA14
artsgRNA15
artsgRNA16
artsgRNA17
artsgRNA18
artsgRNA19
artsgRNA20
artsgRNA21
artsgRNA22
artsgRNA23
artsgRNA24
artsgRNA25
artsgRNA26
artsgRNA27
artsgRNA28
artsgRNA29
artsgRNA30
artsgRNA31
artsgRNA32
artsgRNA33
artsgRNA34
artsgRNA35
artsgRNA36
artsgRNA37

b

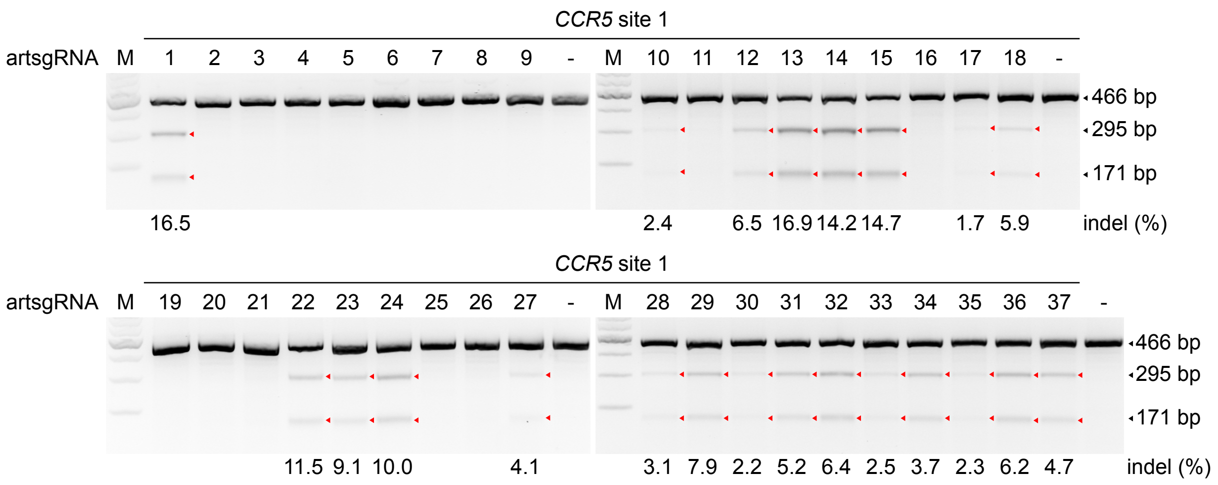

CCR5 site 1
artsgRNA M 1 2 3 4 5 6 7 8 9 - M 10 11 12 13 14 15 16 17 18 -
466 bp
295 bp
171 bp
16.5
2.4
6.5 16.9 14.2 14.7
1.7 5.9
indel (%)
CCR5 site 1
artsgRNA M 19 20 21 22 23 24 25 26 27 - M 28 29 30 31 32 33 34 35 36 37 -
466 bp
295 bp
171 bp
11.5 9.1 10.0
4.1
3.1 7.9 2.2 5.2 6.4 2.5 3.7 2.3 6.2 4.7
indel (%)

c

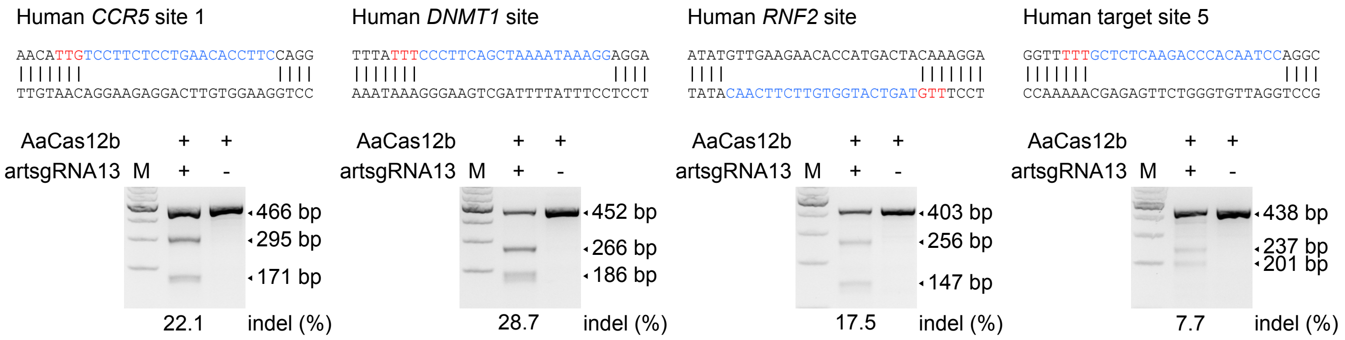

Human CCR5 site 1
AACATTGTCCTTCTCCTGAACACCTTCCAGG
TTGTAACAGGAAGAGGACTTGTGGAAGGTCC
AaCas12b + +
artsgRNA13 M + -
466 bp
295 bp
171 bp
22.1
indel (%)
Human DNMT1 site
TTTATTTCCCTTCAGCTAAAATAAAGGAGGA
AAATAAAGGGAAGTCGATTTTATTTCCTCCT
AaCas12b + +
artsgRNA13 M + -
452 bp
266 bp
186 bp
28.7
indel (%)
Human RNF2 site
ATATGTTGAAGAACACCATGACTACAAAGGA
TATACAACTTCTTGTGGTACTGATGTTTCCT
AaCas12b + +
artsgRNA13 M + -
403 bp
256 bp
147 bp
17.5
indel (%)
Human target site 5
GGTTTTTGCTCTCAAGACCCACAATCCAGGC
CCAAAAACGAGAGTTCTGGGTGTTAGGTCCG
AaCas12b + +
artsgRNA13 M + -
438 bp
237 bp
201 bp
7.7
indel (%)

Figure S8 Artificial sgRNAs directed TcCas12b for genome editing. a Schematic illustrating the secondary structures of the 36 artificial sgRNA (artsgRNA) scaffolds (scaffold: 1 - 12 and 14 - 37). **b** T7EI assay results indicating the genome targeting activity of TcCas12b directed by artsgRNAs in the human 293T cells. Red triangles indicate the cleaved bands. **c** T7EI assay results indicating the simultaneous multiplex genome targeting mediated by AaCas12b combined with artsgRNA13s in human 293T cells.

Supplementary Table 1 Oligonucleotides (oligos) for Cas12b gene synthesis. (see attached XLSX file)

Supplementary Table 2 Oligos for sgRNA scaffold synthesis. (see attached XLSX file)

Supplementary Table 3 Protospacer sequences of human genomic targets. Protospacer targets designed based on type V-B CRISPR-Cas12b locus and type V-A CRISPR-Cas12a locus with their requisite PAMs against different genes in the human genome.

CRISPR-Cas	Gene	Protospacer ID	Protospacer sequences (5′ - 3′)	5′PAM	Strand
Cas12b	*CCR5*	*CCR5*-1	TCCTTCTCCTGAACACCTTC	TTG	+
		CCR5-2	TTTGGCCTGAATAATTGCAG	TTC	+
	DNMT1	*DNMT1*	CCCTTCAGCTAAAATAAAGG	TTT	+
	RNF2	*RNF2*	TAGTCATGGTGTTCTTCAAC	TTG	-
	Intergenic region	Target 5	GCTCTCAAGACCCACAATCC	TTT	+
Cas12a	*CCR5*	*CCR5*-1	TCCTTCTCCTGAACACCTTCCAG	TTG	+
		CCR5-2	GCCTGAATAATTGCAGTAGCTCT	TTTG	+
	DNMT1	*DNMT1*	CCTTCAGCTAAAATAAAGGAGGA	TTTC	+
	RNF2	*RNF2*	GATATGTTGAAGAACACCATGAC	TTTG	+
	Intergenic region	Target 5	CTCTCAAGACCCACAATCCAGGC	TTTG	+

Supplementary Table 4 Oligos for artificial sgRNA (artsgRNA) synthesis. (see attached XLSX file)

Supplementary Table 5 Primers used for T7EI assay in this study.

Primer ID	Primer sequences (5′ – 3′)	Product length (bp)	Cleaved bands (bp)
CCR5-1F<br>CCR5-1R	GTCCTGCCGCTGCTTGTCAT<br>CCCACTTGAGTCCGTGTCACA	466	295 + 171
CCR5-2F<br>CCR5-2R	GCAGCTCTCATTTTCCATACAGT<br>GATCGGGTGTAAACTGAGCTTG	493	277 + 216
DNMT1-F<br>DNMT1-R	CTCCTGCTCGGTGAATTTGG<br>TAGTTGATAAGCGAACCTCACAC	452	270 + 182
RNF2-F<br>RNF2-R	GGAGCTGTAGGCGATTATAGTTGAA<br>TTCTCAAACCCTGGAAAGCACTTT	403	256 + 147
Target5-F<br>Target5-R	ACTTCCACCCTCTGTCTTATCTC<br>CCAGCTTCCTCAAATCTTATGCA	438	237 + 201

Supplementary Sequences

Humanized BhCas12b coding sequence from *Bacillus hisashii* strain C4 (GeneBank ID: NZ_NJGA01000060.1)

ATGGCCACCCGCAGCTTCATCCTGAAGATCGAGCCCAACGAGGAGGTGAAGAAGGGCCTGTG
GAAGACCCACGAGGTGCTGAACCACGGCATCGCCTACTACATGAACATCCTGAAGCTGATCC
GCCAGGAGGCCATCTACGAGCACCACGAGCAGGACCCCAAGAACCCCAAGAAGGTGAGCAAG
GCCGAGATCCAGGCCGAGCTGTGGGACTTCGTGCTGAAGATGCAGAAGTGCAACAGCTTCAC
CCACGAGGTGGACAAGGACGAGGTGTTCAACATCCTGCGCGAGCTGTACGAGGAGCTGGTGC
CCAGCAGCGTGGAGAAGAAGGGCGAGGCCAACCAGCTGAGCAACAAGTTCCTGTACCCCCTG
GTGGACCCCAACAGCCAGAGCGGCAAGGGCACCGCCAGCAGCGGCCGCAAGCCCCGCTGGTA
CAACCTGAAGATCGCCGGCGACCCCAGCTGGGAGGAGGAGAAGAAGAAGTGGGAGGAGGACA
AGAAGAAGGACCCCCTGGCCAAGATCCTGGGCAAGCTGGCCGAGTACGGCCTGATCCCCCTG
TTCATCCCCTACACCGACAGCAACGAGCCCATCGTGAAGGAGATCAAGTGGATGGAGAAGAG
CCGCAACCAGAGCGTGCGCCGCCTGGACAAGGACATGTTCATCCAGGCCCTGGAGCGCTTCC
TGAGCTGGGAGAGCTGGAACCTGAAGGTGAAGGAGGAGTACGAGAAGGTGGAGAAGGAGTAC
AAGACCCTGGAGGAGCGCATCAAGGAGGACATCCAGGCCCTGAAGGCCCTGGAGCAGTACGA
GAAGGAGCGCCAGGAGCAGCTGCTGCGCGACACCCTGAACACCAACGAGTACCGCCTGAGCA
AGCGCGGCCTGCGCGGCTGGCGCGAGATCATCCAGAAGTGGCTGAAGATGGACGAGAACGAG
CCCAGCGAGAAGTACCTGGAGGTGTTCAAGGACTACCAGCGCAAGCACCCCCGCGAGGCCGG
CGACTACAGCGTGTACGAGTTCCTGAGCAAGAAGGAGAACCACTTCATCTGGCGCAACCACC
CCGAGTACCCCTACCTGTACGCCACCTTCTGCGAGATCGACAAGAAGAAGAAGGACGCCAAG
CAGCAGGCCACCTTCACCCTGGCCGACCCCATCAACCACCCCCTGTGGGTGCGCTTCGAGGA
GCGCAGCGGCAGCAACCTGAACAAGTACCGCATCCTGACCGAGCAGCTGCACACCGAGAAGC
TGAAGAAGAAGCTGACCGTGCAGCTGGACCGCCTGATCTACCCCACCGAGAGCGGCGGCTGG
GAGGAGAAGGGCAAGGTGGACATCGTGCTGCTGCCCAGCCGCCAGTTCTACAACCAGATCTT
CCTGGACATCGAGGAGAAGGGCAAGCACGCCTTCACCTACAAGGACGAGAGCATCAAGTTCC
CCCTGAAGGGCACCCTGGGCGGCGCCCGCGTGCAGTTCGACCGCGACCACCTGCGCCGCTAC
CCCCACAAGGTGGAGAGCGGCAACGTGGGCCGCATCTACTTCAACATGACCGTGAACATCGA
GCCCACCGAGAGCCCCGTGAGCAAGAGCCTGAAGATCCACCGCGACGACTTCCCCAAGGTGG
TGAACTTCAAGCCCAAGGAGCTGACCGAGTGGATCAAGGACAGCAAGGGCAAGAAGCTGAAG
AGCGGCATCGAGAGCCTGGAGATCGGCCTGCGCGTGATGAGCATCGACCTGGGCCAGCGCCA
GGCCGCCGCCGCCAGCATCTTCGAGGTGGTGGACCAGAAGCCCGACATCGAGGGCAAGCTGT
TCTTCCCCATCAAGGGCACCGAGCTGTACGCCGTGCACCGCGCCAGCTTCAACATCAAGCTG
CCCGGCGAGACCCTGGTGAAGAGCCGCGAGGTGCTGCGCAAGGCCCGCGAGGACAACCTGAA
GCTGATGAACCAGAAGCTGAACTTCCTGCGCAACGTGCTGCACTTCCAGCAGTTCGAGGACA
TCACCGAGCGCGAGAAGCGCGTGACCAAGTGGATCAGCCGCCAGGAGAACAGCGACGTGCCC
CTGGTGTACCAGGACGAGCTGATCCAGATCCGCGAGCTGATGTACAAGCCCTACAAGGACTG
GGTGGCCTTCCTGAAGCAGCTGCACAAGCGCCTGGAGGTGGAGATCGGCAAGGAGGTGAAGC
ACTGGCGCAAGAGCCTGAGCGACGGCCGCAAGGGCCTGTACGGCATCAGCCTGAAGAACATC
GACGAGATCGACCGCACCCGCAAGTTCCTGCTGCGCTGGAGCCTGCGCCCCACCGAGCCCGG
CGAGGTGCGCCGCCTGGAGCCCGGCCAGCGCTTCGCCATCGACCAGCTGAACCACCTGAACG
CCCTGAAGGAGGACCGCCTGAAGAAGATGGCCAACACCATCATCATGCACGCCCTGGGCTAC

TGCTACGACGTGCGCAAGAAGAAGTGGCAGGCCAAGAACCCCGCCTGCCAGATCATCCTGTT
CGAGGACCTGAGCAACTACAACCCCTACGAGGAGCGCAGCCGCTTCGAGAACAGCAAGCTGA
TGAAGTGGAGCCGCCGCGAGATCCCCCGCCAGGTGGCCCTGCAGGGCGAGATCTACGGCCTG
CAGGTGGGCGAGGTGGGCGCCCAGTTCAGCAGCCGCTTCCACGCCAAGACCGGCAGCCCCGG
CATCCGCTGCAGCGTGGTGACCAAGGAGAAGCTGCAGGACAACCGCTTCTTCAAGAACCTGC
AGCGCGAGGGCCGCCTGACCCTGGACAAGATCGCCGTGCTGAAGGAGGGCGACCTGTACCCC
GACAAGGGCGGCGAGAAGTTCATCAGCCTGAGCAAGGACCGCAAGTGCGTGACCACCCACGC
CGACATCAACGCCGCCCAGAACCTGCAGAAGCGCTTCTGGACCCGCACCCACGGCTTCTACA
AGGTGTACTGCAAGGCCTACCAGGTGGACGGCCAGACCGTGTACATCCCCGAGAGCAAGGAC
CAGAAGCAGAAGATCATCGAGGAGTTCGGCGAGGGCTACTTCATCCTGAAGGACGGCGTGTA
CGAGTGGGTGAACGCCGGCAAGCTGAAGATCAAGAAGGGCAGCAGCAAGCAGAGCAGCAGCG
AGCTGGTGGACAGCGACATCCTGAAGGACAGCTTCGACCTGGCCAGCGAGCTGAAGGGCGAG
AAGCTGATGCTGTACCGCGACCCCAGCGGCAACGTGTTCCCCAGCGACAAGTGGATGGCCGC
CGGCGTGTTCTTCGGCAAGCTGGAGCGCATCCTGATCAGCAAGCTGACCAACCAGTACAGCA
TCAGCACCATCGAGGACGACAGCAGCAAGCAGAGCATG

Humanized Bs3Cas12b coding sequence from *Bacillus sp.* V3-13 contig_40 (GeneBank ID: NZ_PGUZ01000040.1)

ATGGCCATCCGCAGCATCAAGCTGAAGATGAAGACCAACAGCGGCACCGACAGCATCTACCT
GCGCAAGGCCCTGTGGCGCACCCACCAGCTGATCAACGAGGGCATCGCCTACTACATGAACC
TGCTGACCCTGTACCGCCAGGAGGCCATCGGCGACAAGACCAAGGAGGCCTACCAGGCCGAG
CTGATCAACATCATCCGCAACCAGCAGCGCAACAACGGCAGCAGCGAGGAGCACGGCAGCGA
CCAGGAGATCCTGGCCCTGCTGCGCCAGCTGTACGAGCTGATCATCCCCAGCAGCATCGGCG
AGAGCGGCGACGCCAACCAGCTGGGCAACAAGTTCCTGTACCCCCTGGTGGACCCCAACAGC
CAGAGCGGCAAGGGCACCAGCAACGCCGGCCGCAAGCCCCGCTGGAAGCGCCTGAAGGAGGA
GGGCAACCCCGACTGGGAGCTGGAGAAGAAGAAGGACGAGGAGCGCAAGGCCAAGGACCCCA
CCGTGAAGATCTTCGACAACCTGAACAAGTACGGCCTGCTGCCCCTGTTCCCCCTGTTCACC
AACATCCAGAAGGACATCGAGTGGCTGCCCCTGGGCAAGCGCCAGAGCGTGCGCAAGTGGGA
CAAGGACATGTTCATCCAGGCCATCGAGCGCCTGCTGAGCTGGGAGAGCTGGAACCGCCGCG
TGGCCGACGAGTACAAGCAGCTGAAGGAGAAGACCGAGAGCTACTACAAGGAGCACCTGACC
GGCGGCGAGGAGTGGATCGAGAAGATCCGCAAGTTCGAGAAGGAGCGCAACATGGAGCTGGA
GAAGAACGCCTTCGCCCCCAACGACGGCTACTTCATCACCAGCCGCCAGATCCGCGGCTGGG
ACCGCGTGTACGAGAAGTGGAGCAAGCTGCCCGAGAGCGCCAGCCCCGAGGAGCTGTGGAAG
GTGGTGGCCGAGCAGCAGAACAAGATGAGCGAGGGCTTCGGCGACCCCAAGGTGTTCAGCTT
CCTGGCCAACCGCGAGAACCGCGACATCTGGCGCGGCCACAGCGAGCGCATCTACCACATCG
CCGCCTACAACGGCCTGCAGAAGAAGCTGAGCCGCACCAAGGAGCAGGCCACCTTCACCCTG
CCCGACGCCATCGAGCACCCCCTGTGGATCCGCTACGAGAGCCCCGGCGGCACCAACCTGAA
CCTGTTCAAGCTGGAGGAGAAGCAGAAGAAGAACTACTACGTGACCCTGAGCAAGATCATCT
GGCCCAGCGAGGAGAAGTGGATCGAGAAGGAGAACATCGAGATCCCCCTGGCCCCCAGCATC
CAGTTCAACCGCCAGATCAAGCTGAAGCAGCACGTGAAGGGCAAGCAGGAGATCAGCTTCAG
CGACTACAGCAGCCGCATCAGCCTGGACGGCGTGCTGGGCGGCAGCCGCATCCAGTTCAACC
GCAAGTACATCAAGAACCACAAGGAGCTGCTGGGCGAGGGCGACATCGGCCCCGTGTTCTTC
AACCTGGTGGTGGACGTGGCCCCCCTGCAGGAGACCCGCAACGGCCGCCTGCAGAGCCCCAT

CGGCAAGGCCCTGAAGGTGATCAGCAGCGACTTCAGCAAGGTGATCGACTACAAGCCCAAGG
AGCTGATGGACTGGATGAACACCGGCAGCGCCAGCAACAGCTTCGGCGTGGCCAGCCTGCTG
GAGGGCATGCGCGTGATGAGCATCGACATGGGCCAGCGCACCAGCGCCAGCGTGAGCATCTT
CGAGGTGGTGAAGGAGCTGCCCAAGGACCAGGAGCAGAAGCTGTTCTACAGCATCAACGACA
CCGAGCTGTTCGCCATCCACAAGCGCAGCTTCCTGCTGAACCTGCCCGGCGAGGTGGTGACC
AAGAACAACAAGCAGCAGCGCCAGGAGCGCCGCAAGAAGCGCCAGTTCGTGCGCAGCCAGAT
CCGCATGCTGGCCAACGTGCTGCGCCTGGAGACCAAGAAGACCCCCGACGAGCGCAAGAAGG
CCATCCACAAGCTGATGGAGATCGTGCAGAGCTACGACAGCTGGACCGCCAGCCAGAAGGAG
GTGTGGGAGAAGGAGCTGAACCTGCTGACCAACATGGCCGCCTTCAACGACGAGATCTGGAA
GGAGAGCCTGGTGGAGCTGCACCACCGCATCGAGCCCTACGTGGGCCAGATCGTGAGCAAGT
GGCGCAAGGGCCTGAGCGAGGGCCGCAAGAACCTGGCCGGCATCAGCATGTGGAACATCGAC
GAGCTGGAGGACACCCGCCGCCTGCTGATCAGCTGGAGCAAGCGCAGCCGCACCCCCGGCGA
GGCCAACCGCATCGAGACCGACGAGCCCTTCGGCAGCAGCCTGCTGCAGCACATCCAGAACG
TGAAGGACGACCGCCTGAAGCAGATGGCCAACCTGATCATCATGACCGCCCTGGGCTTCAAG
TACGACAAGGAGGAGAAGGACCGCTACAAGCGCTGGAAGGAGACCTACCCCGCCTGCCAGAT
CATCCTGTTCGAGAACCTGAACCGCTACCTGTTCAACCTGGACCGCAGCCGCCGCGAGAACA
GCCGCCTGATGAAGTGGGCCCACCGCAGCATCCCCCGCACCGTGAGCATGCAGGGCGAGATG
TTCGGCCTGCAGGTGGGCGACGTGCGCAGCGAGTACAGCAGCCGCTTCCACGCCAAGACCGG
CGCCCCCGGCATCCGCTGCCACGCCCTGACCGAGGAGGACCTGAAGGCCGGCAGCAACACCC
TGAAGCGCCTGATCGAGGACGGCTTCATCAACGAGAGCGAGCTGGCCTACCTGAAGAAGGGC
GACATCATCCCCAGCCAGGGCGGCGAGCTGTTCGTGACCCTGAGCAAGCGCTACAAGAAGGA
CAGCGACAACAACGAGCTGACCGTGATCCACGCCGACATCAACGCCGCCCAGAACCTGCAGA
AGCGCTTCTGGCAGCAGAACAGCGAGGTGTACCGCGTGCCCTGCCAGCTGGCCCGCATGGGC
GAGGACAAGCTGTACATCCCCAAGAGCCAGACCGAGACCATCAAGAAGTACTTCGGCAAGGG
CAGCTTCGTGAAGAACAACACCGAGCAGGAGGTGTACAAGTGGGAGAAGAGCGAGAAGATGA
AGATCAAGACCGACACCACCTTCGACCTGCAGGACCTGGACGGCTTCGAGGACATCAGCAAG
ACCATCGAGCTGGCCCAGGAGCAGCAGAAGAAGTACCTGACCATGTTCCGCGACCCCAGCGG
CTACTTCTTCAACAACGAGACCTGGCGCCCCCAGAAGGAGTACTGGAGCATCGTGAACAACA
TCATCAAGAGCTGCCTGAAGAAGAAGATCCTGAGCAACAAGGTGGAGCTG

Humanized DiCas12b coding sequence from *Desulfovibrio inopinatus* DSM 10711 (GeneBank: NZ_KE386879.1)

ATGCCCACCCGCACCATCAACCTGAAGCTGGTGCTGGGCAAGAACCCCGAGAACGCCACCCT
GCGCCGCGCCCTGTTCAGCACCCACCGCCTGGTGAACCAGGCCACCAAGCGCATCGAGGAGT
TCCTGCTGCTGTGCCGCGGCGAGGCCTACCGCACCGTGGACAACGAGGGCAAGGAGGCCGAG
ATCCCCCGCCACGCCGTGCAGGAGGAGGCCCTGGCCTTCGCCAAGGCCGCCCAGCGCCACAA
CGGCTGCATCAGCACCTACGAGGACCAGGAGATCCTGGACGTGCTGCGCCAGCTGTACGAGC
GCCTGGTGCCCAGCGTGAACGAGAACAACGAGGCCGGCGACGCCCAGGCCGCCAACGCCTGG
GTGAGCCCCCTGATGAGCGCCGAGAGCGAGGGCGGCCTGAGCGTGTACGACAAGGTGCTGGA
CCCCCCCCCCGTGTGGATGAAGCTGAAGGAGGAGAAGGCCCCCGGCTGGGAGGCCGCCAGCC
AGATCTGGATCCAGAGCGACGAGGGCCAGAGCCTGCTGAACAAGCCCGGCAGCCCCCCCCGC
TGGATCCGCAAGCTGCGCAGCGGCCAGCCCTGGCAGGACGACTTCGTGAGCGACCAGAAGAA
GAAGCAGGACGAGCTGACCAAGGGCAACGCCCCCCTGATCAAGCAGCTGAAGGAGATGGGCC
TGCTGCCCCTGGTGAACCCCTTCTTCCGCCACCTGCTGGACCCCGAGGGCAAGGGCGTGAGC

CCCTGGGACCGCCTGGCCGTGCGCGCCGCCGTGGCCCACTTCATCAGCTGGGAGAGCTGGAA
CCACCGCACCCGCGCCGAGTACAACAGCCTGAAGCTGCGCCGCGACGAGTTCGAGGCCGCCA
GCGACGAGTTCAAGGACGACTTCACCCTGCTGCGCCAGTACGAGGCCAAGCGCCACAGCACC
CTGAAGAGCATCGCCCTGGCCGACGACAGCAACCCCTACCGCATCGGCGTGCGCAGCCTGCG
CGCCTGGAACCGCGTGCGCGAGGAGTGGATCGACAAGGGCGCCACCGAGGAGCAGCGCGTGA
CCATCCTGAGCAAGCTGCAGACCCAGCTGCGCGGCAAGTTCGGCGACCCCGACCTGTTCAAC
TGGCTGGCCCAGGACCGCCACGTGCACCTGTGGAGCCCCCGCGACAGCGTGACCCCCCTGGT
GCGCATCAACGCCGTGGACAAGGTGCTGCGCCGCCGCAAGCCCTACGCCCTGATGACCTTCG
CCCACCCCCGCTTCCACCCCCGCTGGATCCTGTACGAGGCCCCCGGCGGCAGCAACCTGCGC
CAGTACGCCCTGGACTGCACCGAGAACGCCCTGCACATCACCCTGCCCCTGCTGGTGGACGA
CGCCCACGGCACCTGGATCGAGAAGAAGATCCGCGTGCCCCTGGCCCCCAGCGGCCAGATCC
AGGACCTGACCCTGGAGAAGCTGGAGAAGAAGAAGAACCGCCTGTACTACCGCAGCGGCTTC
CAGCAGTTCGCCGGCCTGGCCGGCGGCGCCGAGGTGCTGTTCCACCGCCCCTACATGGAGCA
CGACGAGCGCAGCGAGGAGAGCCTGCTGGAGCGCCCCGGCGCCGTGTGGTTCAAGCTGACCC
TGGACGTGGCCACCCAGGCCCCCCCCAACTGGCTGGACGGCAAGGGCCGCGTGCGCACCCCC
CCCGAGGTGCACCACTTCAAGACCGCCCTGAGCAACAAGAGCAAGCACACCCGCACCCTGCA
GCCCGGCCTGCGCGTGCTGAGCGTGGACCTGGGCATGCGCACCTTCGCCAGCTGCAGCGTGT
TCGAGCTGATCGAGGGCAAGCCCGAGACCGGCCGCGCCTTCCCCGTGGCCGACGAGCGCAGC
ATGGACAGCCCCAACAAGCTGTGGGCCAAGCACGAGCGCAGCTTCAAGCTGACCCTGCCCGG
CGAGACCCCCAGCCGCAAGGAGGAGGAGGAGCGCAGCATCGCCCGCGCCGAGATCTACGCCC
TGAAGCGCGACATCCAGCGCCTGAAGAGCCTGCTGCGCCTGGGCGAGGAGGACAACGACAAC
CGCCGCGACGCCCTGCTGGAGCAGTTCTTCAAGGGCTGGGGCGAGGAGGACGTGGTGCCCGG
CCAGGCCTTCCCCCGCAGCCTGTTCCAGGGCCTGGGCGCCGCCCCCTTCCGCAGCACCCCCG
AGCTGTGGCGCCAGCACTGCCAGACCTACTACGACAAGGCCGAGGCCTGCCTGGCCAAGCAC
ATCAGCGACTGGCGCAAGCGCACCCGCCCCCGCCCCACCAGCCGCGAGATGTGGTACAAGAC
CCGCAGCTACCACGGCGGCAAGAGCATCTGGATGCTGGAGTACCTGGACGCCGTGCGCAAGC
TGCTGCTGAGCTGGAGCCTGCGCGGCCGCACCTACGGCGCCATCAACCGCCAGGACACCGCC
CGCTTCGGCAGCCTGGCCAGCCGCCTGCTGCACCACATCAACAGCCTGAAGGAGGACCGCAT
CAAGACCGGCGCCGACAGCATCGTGCAGGCCGCCCGCGGCTACATCCCCCTGCCCCACGGCA
AGGGCTGGGAGCAGCGCTACGAGCCCTGCCAGCTGATCCTGTTCGAGGACCTGGCCCGCTAC
CGCTTCCGCGTGGACCGCCCCCGCCGCGAGAACAGCCAGCTGATGCAGTGGAACCACCGCGC
CATCGTGGCCGAGACCACCATGCAGGCCGAGCTGTACGGCCAGATCGTGGAGAACACCGCCG
CCGGCTTCAGCAGCCGCTTCCACGCCGCCACCGGCGCCCCCGGCGTGCGCTGCCGCTTCCTG
CTGGAGCGCGACTTCGACAACGACCTGCCCAAGCCCTACCTGCTGCGCGAGCTGAGCTGGAT
GCTGGGCAACACCAAGGTGGAGAGCGAGGAGGAGAAGCTGCGCCTGCTGAGCGAGAAGATCC
GCCCCGGCAGCCTGGTGCCCTGGGACGGCGGCGAGCAGTTCGCCACCCTGCACCCCAAGCGC
CAGACCCTGTGCGTGATCCACGCCGACATGAACGCCGCCCAGAACCTGCAGCGCCGCTTCTT
CGGCCGCTGCGGCGAGGCCTTCCGCCTGGTGTGCCAGCCCCACGGCGACGACGTGCTGCGCC
TGGCCAGCACCCCCGGCGCCCGCCTGCTGGGCGCCCTGCAGCAGCTGGAGAACGGCCAGGGC
GCCTTCGAGCTGGTGCGCGACATGGGCAGCACCAGCCAGATGAACCGCTTCGTGATGAAGAG
CCTGGGCAAGAAGAAGATCAAGCCCCTGCAGGACAACAACGGCGACGACGAGCTGGAGGACG
TGCTGAGCGTGCTGCCCGAGGAGGACGACACCGGCCGCATCACCGTGTTCCGCGACAGCAGC
GGCATCTTCTTCCCCTGCAACGTGTGGATCCCCGCCAAGCAGTTCTGGCCCGCCGTGCGCGC
CATGATCTGGAAGGTGATGGCCAGCCACAGCCTGGGC

Humanized LsCas12b coding sequence from *Laceyella sediminis* strain RHA1 (GeneBank ID: NZ_PVTZ01000002.1)

ATGAGCATCCGCAGCTTCAAGCTGAAGATCAAGACCAAGAGCGGCGTGAACGCCGAGGAGCT
GCGCCGCGGCCTGTGGCGCACCCACCAGCTGATCAACGACGGCATCGCCTACTACATGAACT
GGCTGGTGCTGCTGCGCCAGGAGGACCTGTTCATCCGCAACGAGGAGACCAACGAGATCGAG
AAGCGCAGCAAGGAGGAGATCCAGGGCGAGCTGCTGGAGCGCGTGCACAAGCAGCAGCAGCG
CAACCAGTGGAGCGGCGAGGTGGACGACCAGACCCTGCTGCAGACCCTGCGCCACCTGTACG
AGGAGATCGTGCCCAGCGTGATCGGCAAGAGCGGCAACGCCAGCCTGAAGGCCCGCTTCTTC
CTGGGCCCCCTGGTGGACCCCAACAACAAGACCACCAAGGACGTGAGCAAGAGCGGCCCCAC
CCCCAAGTGGAAGAAGATGAAGGACGCCGGCGACCCCAACTGGGTGCAGGAGTACGAGAAGT
ACATGGCCGAGCGCCAGACCCTGGTGCGCCTGGAGGAGATGGGCCTGATCCCCCTGTTCCCC
ATGTACACCGACGAGGTGGGCGACATCCACTGGCTGCCCCAGGCCAGCGGCTACACCCGCAC
CTGGGACCGCGACATGTTCCAGCAGGCCATCGAGCGCCTGCTGAGCTGGGAGAGCTGGAACC
GCCGCGTGCGCGAGCGCCGCGCCCAGTTCGAGAAGAAGACCCACGACTTCGCCAGCCGCTTC
AGCGAGAGCGACGTGCAGTGGATGAACAAGCTGCGCGAGTACGAGGCCCAGCAGGAGAAGAG
CCTGGAGGAGAACGCCTTCGCCCCCAACGAGCCCTACGCCCTGACCAAGAAGGCCCTGCGCG
GCTGGGAGCGCGTGTACCACAGCTGGATGCGCCTGGACAGCGCCGCCAGCGAGGAGGCCTAC
TGGCAGGAGGTGGCCACCTGCCAGACCGCCATGCGCGGCGAGTTCGGCGACCCCGCCATCTA
CCAGTTCCTGGCCCAGAAGGAGAACCACGACATCTGGCGCGGCTACCCCGAGCGCGTGATCG
ACTTCGCCGAGCTGAACCACCTGCAGCGCGAGCTGCGCCGCGCCAAGGAGGACGCCACCTTC
ACCCTGCCCGACAGCGTGGACCACCCCCTGTGGGTGCGCTACGAGGCCCCCGGCGGCACCAA
CATCCACGGCTACGACCTGGTGCAGGACACCAAGCGCAACCTGACCCTGATCCTGGACAAGT
TCATCCTGCCCGACGAGAACGGCAGCTGGCACGAGGTGAAGAAGGTGCCCTTCAGCCTGGCC
AAGAGCAAGCAGTTCCACCGCCAGGTGTGGCTGCAGGAGGAGCAGAAGCAGAAGAAGCGCGA
GGTGGTGTTCTACGACTACAGCACCAACCTGCCCCACCTGGGCACCCTGGCCGGCGCCAAGC
TGCAGTGGGACCGCAACTTCCTGAACAAGCGCACCCAGCAGCAGATCGAGGAGACCGGCGAG
ATCGGCAAGGTGTTCTTCAACATCAGCGTGGACGTGCGCCCCGCCGTGGAGGTGAAGAACGG
CCGCCTGCAGAACGGCCTGGGCAAGGCCCTGACCGTGCTGACCCACCCCGACGGCACCAAGA
TCGTGACCGGCTGGAAGGCCGAGCAGCTGGAGAAGTGGGTGGGCGAGAGCGGCCGCGTGAGC
AGCCTGGGCCTGGACAGCCTGAGCGAGGGCCTGCGCGTGATGAGCATCGACCTGGGCCAGCG
CACCAGCGCCACCGTGAGCGTGTTCGAGATCACCAAGGAGGCCCCCGACAACCCCTACAAGT
TCTTCTACCAGCTGGAGGGCACCGAGCTGTTCGCCGTGCACCAGCGCAGCTTCCTGCTGGCC
CTGCCCGGCGAGAACCCCCCCCAGAAGATCAAGCAGATGCGCGAGATCCGCTGGAAGGAGCG
CAACCGCATCAAGCAGCAGGTGGACCAGCTGAGCGCCATCCTGCGCCTGCACAAGAAGGTGA
ACGAGGACGAGCGCATCCAGGCCATCGACAAGCTGCTGCAGAAGGTGGCCAGCTGGCAGCTG
AACGAGGAGATCGCCACCGCCTGGAACCAGGCCCTGAGCCAGCTGTACAGCAAGGCCAAGGA
GAACGACCTGCAGTGGAACCAGGCCATCAAGAACGCCCACCACCAGCTGGAGCCCGTGGTGG
GCAAGCAGATCAGCCTGTGGCGCAAGGACCTGAGCACCGGCCGCCAGGGCATCGCCGGCCTG
AGCCTGTGGAGCATCGAGGAGCTGGAGGCCACCAAGAAGCTGCTGACCCGCTGGAGCAAGCG
CAGCCGCGAGCCCGGCGTGGTGAAGCGCATCGAGCGCTTCGAGACCTTCGCCAAGCAGATCC
AGCACCACATCAACCAGGTGAAGGAGAACCGCCTGAAGCAGCTGGCCAACCTGATCGTGATG
ACCGCCCTGGGCTACAAGTACGACCAGGAGCAGAAGAAGTGGATCGAGGTGTACCCCGCCTG

CCAGGTGGTGCTGTTCGAGAACCTGCGCAGCTACCGCTTCAGCTACGAGCGCAGCCGCCGCG
AGAACAAGAAGCTGATGGAGTGGAGCCACCGCAGCATCCCCAAGCTGGTGCAGATGCAGGGC
GAGCTGTTCGGCCTGCAGGTGGCCGACGTGTACGCCGCCTACAGCAGCCGCTACCACGGCCG
CACCGGCGCCCCCGGCATCCGCTGCCACGCCCTGACCGAGGCCGACCTGCGCAACGAGACCA
ACATCATCCACGAGCTGATCGAGGCCGGCTTCATCAAGGAGGAGCACCGCCCCTACCTGCAG
CAGGGCGACCTGGTGCCCTGGAGCGGCGGCGAGCTGTTCGCCACCCTGCAGAAGCCCTACGA
CAACCCCCGCATCCTGACCCTGCACGCCGACATCAACGCCGCCCAGAACATCCAGAAGCGCT
TCTGGCACCCCAGCATGTGGTTCCGCGTGAACTGCGAGAGCGTGATGGAGGGCGAGATCGTG
ACCTACGTGCCCAAGAACAAGACCGTGCACAAGAAGCAGGGCAAGACCTTCCGCTTCGTGAA
GGTGGAGGGCAGCGACGTGTACGAGTGGGCCAAGTGGAGCAAGAACCGCAACAAGAACACCT
TCAGCAGCATCACCGAGCGCAAGCCCCCCAGCAGCATGATCCTGTTCCGCGACCCCAGCGGC
ACCTTCTTCAAGGAGCAGGAGTGGGTGGAGCAGAAGACCTTCTGGGGCAAGGTGCAGAGCAT
GATCCAGGCCTACATGAAGAAGACCATCGTGCAGCGCATGGAGGAG

Humanized SbCas12b coding sequence from *Spirochaetes bacterium* GWB1_27_13 (GeneBank ID: MIAN01000063.1)

ATGAGCTTCACCATCAGCTACCCCTTCAAGCTGATCATCAAGAACAAGGACGAGGCCAAGGC
CCTGCTGGACACCCACCAGTACATGAACGAGGGCGTGAAGTACTACCTGGAGAAGCTGCTGA
TGTTCCGCCAGGAGAAGATCTTCATCGGCGAGGACGAGACCGGCAAGCGCATCTACATCGAG
GAGACCGAGTACAAGAAGCAGATCGAGGAGTTCTACCTGATCAAGAAGACCGAGCTGGGCCG
CAACCTGACCCTGACCCTGGACGAGTTCAAGACCCTGATGCGCGAGCTGTACATCTGCCTGG
TGAGCAGCAGCATGGAGAACAAGAAGGGCTTCCCCAACGCCCAGCAGGCCAGCCTGAACATC
TTCAGCCCCCTGTTCGACGCCGAGAGCAAGGGCTACATCCTGAAGGAGGAGAACAACAACAT
CAGCCTGATCCACAAGGACTACGGCAAGATCCTGCTGAAGCGCCTGCGCGACAACAACCTGA
TCCCCATCTTCACCAAGTTCACCGACATCAAGAAGATCACCGCCAAGCTGAGCCCCACCGCC
CTGGACCGCATGATCTTCGCCCAGGCCATCGAGAAGCTGCTGAGCTACGAGAGCTGGTGCAA
GCTGATGATCAAGGAGCGCTTCGACAAGGAGGTGAAGATCAAGGAGCTGGAGAACAAGTGCG
AGAACAAGCAGGAGCGCGACAAGATCTTCGAGATCCTGGAGAAGTACGAGGAGGAGCGCCAG
AAGACCTTCGAGCAGGACAGCGGCTTCGCCAAGAAGGGCAAGTTCTACATCACCGGCCGCAT
GCTGAAGGGCTTCGACGAGATCAAGGAGAAGTGGCTGAAGGAGAAGGACCGCAGCGAGCAGA
ACCTGATCAACATCCTGAACAAGTACCAGACCGACAACAGCAAGCTGGTGGGCGACCGCAAC
CTGTTCGAGTTCATCATCAAGCTGGAGAACCAGTGCCTGTGGAACGGCGACATCGACTACCT
GAAGATCAAGCGCGACATCAACAAGAACCAGATCTGGCTGGACCGCCCCGAGATGCCCCGCT
TCACCATGCCCGACTTCAAGAAGCACCCCCTGTGGTACCGCTACGAGGACCCCAGCAACAGC
AACTTCCGCAACTACAAGATCGAGGTGGTGAAGGACGAGAACTACATCACCATCCCCCTGAT
CACCGAGCGCAACAACGAGTACTTCGAGGAGAACTACACCTTCAACCTGGCCAAGCTGAAGA
AGCTGAGCGAGAACATCACCTTCATCCCCAAGAGCAAGAACAAGGAGTTCGAGTTCATCGAC
AGCAACGACGAGGAGGAGGACAAGAAGGACCAGAAGAAGAGCAAGCAGTACATCAAGTACTG
CGACACCGCCAAGAACACCAGCTACGGCAAGAGCGGCGGCATCCGCCTGTACTTCAACCGCA
ACGAGCTGGAGAACTACAAGGACGGCAAGAAGATGGACAGCTACACCGTGTTCACCCTGAGC
ATCCGCGACTACAAGAGCCTGTTCGCCAAGGAGAAGCTGCAGCCCCAGATCTTCAACACCGT
GGACAACAAGATCACCAGCCTGAAGATCCAGAAGAAGTTCGGCAACGAGGAGCAGACCAACT
TCCTGAGCTACTTCACCCAGAACCAGATCACCAAGAAGGACTGGATGGACGAGAAGACCTTC

CAGAACGTGAAGGAGCTGAACGAGGGCATCCGCGTGCTGAGCGTGGACCTGGGCCAGCGCTT
CTTCGCCGCCGTGAGCTGCTTCGAGATCATGAGCGAGATCGACAACAACAAGCTGTTCTTCA
ACCTGAACGACCAGAACCACAAGATCATCCGCATCAACGACAAGAACTACTACGCCAAGCAC
ATCTACAGCAAGACCATCAAGCTGAGCGGCGAGGACGACGACCTGTACAAGGAGCGCAAGAT
CAACAAGAACTACAAGCTGAGCTACCAGGAGCGCAAGAACAAGATCGGCATCTTCACCCGCC
AGATCAACAAGCTGAACCAGCTGCTGAAGATCATCCGCAACGACGAGATCGACAAGGAGAAG
TTCAAGGAGCTGATCGAGACCACCAAGCGCTACGTGAAGAACACCTACAACGACGGCATCAT
CGACTGGAACAACGTGGACAACAAGATCCTGAGCTACGAGAACAAGGAGGACGTGATCAACC
TGCACAAGGAGCTGGACAAGAAGCTGGAGATCGACTTCAAGGAGTTCATCCGCGAGTGCCGC
AAGCCCATCTTCCGCAGCGGCGGCCTGAGCATGCAGCGCATCGACTTCCTGGAGAAGCTGAA
CAAGCTGAAGCGCAAGTGGGTGGCCCGCACCCAGAAGAGCGCCGAGAGCATCGTGCTGACCC
CCAAGTTCGGCTACAAGCTGAAGGAGCACATCAACGAGCTGAAGGACAACCGCGTGAAGCAG
GGCGTGAACTACATCCTGATGACCGCCCTGGGCTACATCAAGGACAACGAGATCAAGAACGA
CAGCAAGAAGAAGCAGAAGGAGGACTGGGTGAAGAAGAACCGCGCCTGCCAGATCATCCTGA
TGGAGAAGCTGACCGAGTACACCTTCGCCGAGGACCGCCCCCGCGAGGAGAACAGCAAGCTG
CGCATGTGGAGCCACCGCCAGATCTTCAACTTCCTGCAGCAGAAGGCCAGCCTGTGGGGCAT
CCTGGTGGGCGACGTGTTCGCCCCCTACACCAGCAAGTGCCTGAGCGACAACAACGCCCCCG
GCATCCGCTGCCACCAGGTGACCAAGAAGGACCTGATCGACAACAGCTGGTTCCTGAAGATC
GTGGTGAAGGACGACGCCTTCTGCGACCTGATCGAGATCAACAAGGAGAACGTGAAGAACAA
GAGCATCAAGATCAACGACATCCTGCCCCTGCGCGGCGGCGAGCTGTTCGCCAGCATCAAGG
ACGGCAAGCTGCACATCGTGCAGGCCGACATCAACGCCAGCCGCAACATCGCCAAGCGCTTC
CTGAGCCAGATCAACCCCTTCCGCGTGGTGCTGAAGAAGGACAAGGACGAGACCTTCCACCT
GAAGAACGAGCCCAACTACCTGAAGAACTACTACAGCATCCTGAACTTCGTGCCCACCAACG
AGGAGCTGACCTTCTTCAAGGTGGAGGAGAACAAGGACATCAAGCCCACCAAGCGCATCAAG
ATGGACAAGCACGAGAAGGAGAGCACCGACGAGGGCGACGACTACAGCAAGAACCAGATCGC
CCTGTTCCGCGACGACAGCGGCATCTTCTTCGACAAGAGCCTGTGGGTGGACGGCAAGATCT
TCTGGAGCGTGGTGAAGAACAAGATGACCAAGCTGCTGCGCGAGCGCAACAACAAGAAGAAC
GGCAGCAAG

Humanized TcCas12b coding sequence from *Tuberibacillus calidus* DSM 17572 (GeneBank ID: NZ_KE387196.1)

ATGAACATCCACCTGAAGGAGCTGATCCGCATGGCCACCAAGAGCTTCATCCTGAAGATGAA
GACCAAGAACAACCCCCAGCTGCGCCTGAGCCTGTGGAAGACCCACGAGCTGTTCAACTTCG
GCGTGGCCTACTACATGGACCTGCTGAGCCTGTTCCGCCAGAAGGACCTGTACATGCACAAC
GACGAGGACCCCGACCACCCCGTGGTGCTGAAGAAGGAGGAGATCCAGGAGCGCCTGTGGAT
GAAGGTGCGCGAGACCCAGCAGAAGAACGGCTTCCACGGCGAGGTGAGCAAGGACGAGGTGC
TGGAGACCCTGCGCGCCCTGTACGAGGAGCTGGTGCCCAGCGCCGTGGGCAAGAGCGGCGAG
GCCAACCAGATCAGCAACAAGTACCTGTACCCCCTGACCGACCCCGCCAGCCAGAGCGGCAA
GGGCACCGCCAACAGCGGCCGCAAGCCCCGCTGGAAGAAGCTGAAGGAGGCCGGCGACCCCA
GCTGGAAGGACGCCTACGAGAAGTGGGAGAAGGAGCGCCAGGAGGACCCCAAGCTGAAGATC
CTGGCCGCCCTGCAGAGCTTCGGCCTGATCCCCCTGTTCCGCCCCTTCACCGAGAACGACCA
CAAGGCCGTGATCAGCGTGAAGTGGATGCCCAAGAGCAAGAACCAGAGCGTGCGCAAGTTCG
ACAAGGACATGTTCAACCAGGCCATCGAGCGCTTCCTGAGCTGGGAGAGCTGGAACGAGAAG
GTGGCCGAGGACTACGAGAAGACCGTGAGCATCTACGAGAGCCTGCAGAAGGAGCTGAAGGG

CATCAGCACCAAGGCCTTCGAGATCATGGAGCGCGTGGAGAAGGCCTACGAGGCCCACCTGC
GCGAGATCACCTTCAGCAACAGCACCTACCGCATCGGCAACCGCGCCATCCGCGGCTGGACC
GAGATCGTGAAGAAGTGGATGAAGCTGGACCCCAGCGCCCCCCAGGGCAACTACCTGGACGT
GGTGAAGGACTACCAGCGCCGCCACCCCCGCGAGAGCGGCGACTTCAAGCTGTTCGAGCTGC
TGAGCCGCCCCGAGAACCAGGCCGCCTGGCGCGAGTACCCCGAGTTCCTGCCCCTGTACGTG
AAGTACCGCCACGCCGAGCAGCGCATGAAGACCGCCAAGAAGCAGGCCACCTTCACCCTGTG
CGACCCCATCCGCCACCCCCTGTGGGTGCGCTACGAGGAGCGCAGCGGCACCAACCTGAACA
AGTACCGCCTGATCATGAACGAGAAGGAGAAGGTGGTGCAGTTCGACCGCCTGATCTGCCTG
AACGCCGACGGCCACTACGAGGAGCAGGAGGACGTGACCGTGCCCCTGGCCCCCAGCCAGCA
GTTCGACGACCAGATCAAGTTCAGCAGCGAGGACACCGGCAAGGGCAAGCACAACTTCAGCT
ACTACCACAAGGGCATCAACTACGAGCTGAAGGGCACCCTGGGCGGCGCCCGCATCCAGTTC
GACCGCGAGCACCTGCTGCGCCGCCAGGGCGTGAAGGCCGGCAACGTGGGCCGCATCTTCCT
GAACGTGACCCTGAACATCGAGCCCATGCAGCCCTTCAGCCGCAGCGGCAACCTGCAGACCA
GCGTGGGCAAGGCCCTGAAGGTGTACGTGGACGGCTACCCCAAGGTGGTGAACTTCAAGCCC
AAGGAGCTGACCGAGCACATCAAGGAGAGCGAGAAGAACACCCTGACCCTGGGCGTGGAGAG
CCTGCCCACCGGCCTGCGCGTGATGAGCGTGGACCTGGGCCAGCGCCAGGCCGCCGCCATCA
GCATCTTCGAGGTGGTGAGCGAGAAGCCCGACGACAACAAGCTGTTCTACCCCGTGAAGGAC
ACCGACCTGTTCGCCGTGCACCGCACCAGCTTCAACATCAAGCTGCCCGGCGAGAAGCGCAC
CGAGCGCCGCATGCTGGAGCAGCAGAAGCGCGACCAGGCCATCCGCGACCTGAGCCGCAAGC
TGAAGTTCCTGAAGAACGTGCTGAACATGCAGAAGCTGGAGAAGACCGACGAGCGCGAGAAG
CGCGTGAACCGCTGGATCAAGGACCGCGAGCGCGAGGAGGAGAACCCCGTGTACGTGCAGGA
GTTCGAGATGATCAGCAAGGTGCTGTACAGCCCCCACAGCGTGTGGGTGGACCAGCTGAAGA
GCATCCACCGCAAGCTGGAGGAGCAGCTGGGCAAGGAGATCAGCAAGTGGCGCCAGAGCATC
AGCCAGGGCCGCCAGGGCGTGTACGGCATCAGCCTGAAGAACATCGAGGACATCGAGAAGAC
CCGCCGCCTGCTGTTCCGCTGGAGCATGCGCCCCGAGAACCCCGGCGAGGTGAAGCAGCTGC
AGCCCGGCGAGCGCTTCGCCATCGACCAGCAGAACCACCTGAACCACCTGAAGGACGACCGC
ATCAAGAAGCTGGCCAACCAGATCGTGATGACCGCCCTGGGCTACCGCTACGACGGCAAGCG
CAAGAAGTGGATCGCCAAGCACCCCGCCTGCCAGCTGGTGCTGTTCGAGGACCTGAGCCGCT
ACGCCTTCTACGACGAGCGCAGCCGCCTGGAGAACCGCAACCTGATGCGCTGGAGCCGCCGC
GAGATCCCCAAGCAGGTGGCCCAGATCGGCGGCCTGTACGGCCTGCTGGTGGGCGAGGTGGG
CGCCCAGTACAGCAGCCGCTTCCACGCCAAGAGCGGCGCCCCCGGCATCCGCTGCCGCGTGG
TGAAGGAGCACGAGCTGTACATCACCGAGGGCGGCCAGAAGGTGCGCAACCAGAAGTTCCTG
GACAGCCTGGTGGAGAACAACATCATCGAGCCCGACGACGCCCGCCGCCTGGAGCCCGGCGA
CCTGATCCGCGACCAGGGCGGCGACAAGTTCGCCACCCTGGACGAGCGCGGCGAGCTGGTGA
TCACCCACGCCGACATCAACGCCGCCCAGAACCTGCAGAAGCGCTTCTGGACCCGCACCCAC
GGCCTGTACCGCATCCGCTGCGAGAGCCGCGAGATCAAGGACGCCGTGGTGCTGGTGCCCAG
CGACAAGGACCAGAAGGAGAAGATGGAGAACCTGTTCGGCATCGGCTACCTGCAGCCCTTCA
AGCAGGAGAACGACGTGTACAAGTGGGTGAAGGGCGAGAAGATCAAGGGCAAGAAGACCAGC
AGCCAGAGCGACGACAAGGAGCTGGTGAGCGAGATCCTGCAGGAGGCGAGCGTGATGGCCGA
CGAGCTGAAGGGCAACCGCAAGACCCTGTTCCGCGACCCCAGCGGCTACGTGTTCCCCAAGG
ACCGCTGGTACACCGGCGGCCGCTACTTCGGCACCCTGGAGCACCTGCTGAAGCGCAAGCTG
GCCGAGCGCCGCCTGTTCGACGGCGGCAGCAGCCGCCGCGGCCTGTTCAACGGCACCGACAG
CAACACCAACGTGGAG

pUC19-U6-BhsgRNA partial sequence

(U6-BhsgRNA1_scaffold-*BasI*-*BasI*-terminator)

TGTAAAACGACGGCCAGTGAATTCGAGGGCCTATTTCCCATGATTCCTTCATATTTGCATAT
ACGATACAAGGCTGTTAGAGAGATAATTGGAATTAATTTGACTGTAAACACAAAGATATTAG
TACAAAATACGTGACGTAGAAAGTAATAATTTCTTGGGTAGTTTGCAGTTTTAAAATTATGT
TTTAAAATGGACTATCATATGCTTACCGTAACTTGAAAGTATTTCGATTTCTTGGCTTTATA
TATCTTGTGGAAAGGACGAAACACCGGGAGGTTCTGTCTTTTGGTCAGGACAACCGTCTAGC
TATAAGTGCTGCAGGGTGTGAGAAACTCCTATTGCTGGACGATGTCTCTTACGAGGCATTAG
CACA*GAGACC*GAGAGAG*GGTCTC*AttttttttAAGCTTGGCGTAATCATGGTCATAGCTGTT
TCCTG

pUC19-U6-Bs3sgRNA partial sequence

(U6-BssgRNA1_scaffold-*BasI*-*BasI*-terminator)

TGTAAAACGACGGCCAGTGAATTCGAGGGCCTATTTCCCATGATTCCTTCATATTTGCATAT
ACGATACAAGGCTGTTAGAGAGATAATTGGAATTAATTTGACTGTAAACACAAAGATATTAG
TACAAAATACGTGACGTAGAAAGTAATAATTTCTTGGGTAGTTTGCAGTTTTAAAATTATGT
TTTAAAATGGACTATCATATGCTTACCGTAACTTGAAAGTATTTCGATTTCTTGGCTTTATA
TATCTTGTGGAAAGGACGAAACACCGGGGTGACCTATAGGGTCAATGAATCTGTGCGTGTGC
CATAAGTAATTAAAAATTACCCACCACAGGATTATCTTATTTCTGCTAAGTGTTTAGTTGCC
TGAATACTTAGCAGAAATAATGATGATTGGCACA*GAGACC*GAGAGAG*GGTCTC*Atttttttt
AAGCTTGGCGTAATCATGGTCATAGCTGTTTCCTG

pUC19-U6-LssgRNA partial sequence

(U6-BssgRNA1_scaffold-*BasI*-*BasI*-terminator)

TGTAAAACGACGGCCAGTGAATTCGAGGGCCTATTTCCCATGATTCCTTCATATTTGCATAT
ACGATACAAGGCTGTTAGAGAGATAATTGGAATTAATTTGACTGTAAACACAAAGATATTAG
TACAAAATACGTGACGTAGAAAGTAATAATTTCTTGGGTAGTTTGCAGTTTTAAAATTATGT
TTTAAAATGGACTATCATATGCTTACCGTAACTTGAAAGTATTTCGATTTCTTGGCTTTATA
TATCTTGTGGAAAGGACGAAACACCGGGGCAAAGAATACTGTGCGTGTGCTAAGGATGGAAA
AAATCCATTCAACCACAGGATTACATTATTTATCTAATCACTTAAATCTTTAAGTGATTAGA
TGAATTAAATGTGATTAGCACA*GAGACC*GAGAGAG*GGTCTC*AttttttttAAGCTTGGCGTA
ATCATGGTCATAGCTGTTTCCTG

pUC19-U6-SbsgRNA partial sequence

(U6-BssgRNA1_scaffold-*BasI*-*BasI*-terminator)

TGTAAAACGACGGCCAGTGAATTCGAGGGCCTATTTCCCATGATTCCTTCATATTTGCATAT
ACGATACAAGGCTGTTAGAGAGATAATTGGAATTAATTTGACTGTAAACACAAAGATATTAG
TACAAAATACGTGACGTAGAAAGTAATAATTTCTTGGGTAGTTTGCAGTTTTAAAATTATGT
TTTAAAATGGACTATCATATGCTTACCGTAACTTGAAAGTATTTCGATTTCTTGGCTTTATA
TATCTTGTGGAAAGGACGAAACACCGGGTCTTAGGGTATATCCCAAATTTGTCTTAGTATGT

GCATTGCTTACAGCGACAACTAAGGTTTGTTTATCTTTTTTTTACATTGTAAGATGTTTTAC
ATTATAAAAAGAAGATAATCTTATTGCACA*GAGACC*GAGAGAG*GGTCTC*AttttttttAAGC
TTGGCGTAATCATGGTCATAGCTGTTTCCTG
